# Dissecting disgust and fear in chimpanzees: From facilitation to disruption of cognitive processes

**DOI:** 10.1016/j.isci.2026.116898

**Published:** 2026-07-29

**Authors:** Cécile Sarabian, Andrew J.J. MacIntosh, André Gonçalves, Nobuyuki Kawai, Ikuma Adachi

**Affiliations:** 1 Biosciences Department, Swansea University, Swansea, Wales SA2 8PP, UK; 2 Institute for Advanced Study in Toulouse, Toulouse School of Economics, Université Toulouse Capitole, 31 080 Toulouse, Occitanie, France; 3 Center for the Evolutionary Origins of Human Behavior, Kyoto University, Inuyama, Aichi 484-8506, Japan; 4 Wildlife Research Center, Kyoto University, Inuyama Campus, Inuyama, Aichi 484-8506, Japan; 5 The Wilder Institute, Calgary Zoo, Calgary, Alberta T2E 7V6, Canada; 6 Indianapolis Zoological Society, Indianapolis, IN 46222, USA; 7 Department of Cognitive and Psychological Sciences, Nagoya University, Nagoya, Aichi 464-8601, Japan

**Keywords:** pathogen avoidance, predation risk, threat perception, attention, death, snakes, touch screen experiment, eye-tracking, ape cognition

## Abstract

Disgust and fear are emotions that evolved to address different types of threats: pathogens and predators, respectively. Yet the cognitive mechanisms underlying disgust remain poorly understood, particularly in non-human animals. We investigated how pathogen- and predator-related stimuli influence cognition in captive chimpanzees (*Pan troglodytes*), using touchscreen and eye-tracking tasks. In three experiments, subjects performed a number ordering task while exposed to images (e.g., carcasses, rotten food, invertebrates, snakes) or odors (e.g., of organic decomposition, emesis). Pathogen-related stimuli, particularly those linked to organic decomposition and fermentation, modulated performance in distinct temporal patterns, reducing accuracy and later disrupting efficiency upon re-exposure. In contrast, predator images enhanced performance by reducing response latencies. A fourth eye-tracking experiment further showed that both carcass and snake images captured and held attention. Overall, our results suggest distinct cognitive signatures for pathogen-versus predator-related threats in chimpanzees, offering insight into the evolution of defensive emotions.

## Highlights

• Chimpanzees performed cognitive tasks using touchscreens and eye-tracking
• Pathogen-related images and odors reduced accuracy and later slowed responses
• Snake images improved performance by speeding responses
• Both pathogen- and predator-related images captured and held visual attention

## Graphical abstract

**Figure:**
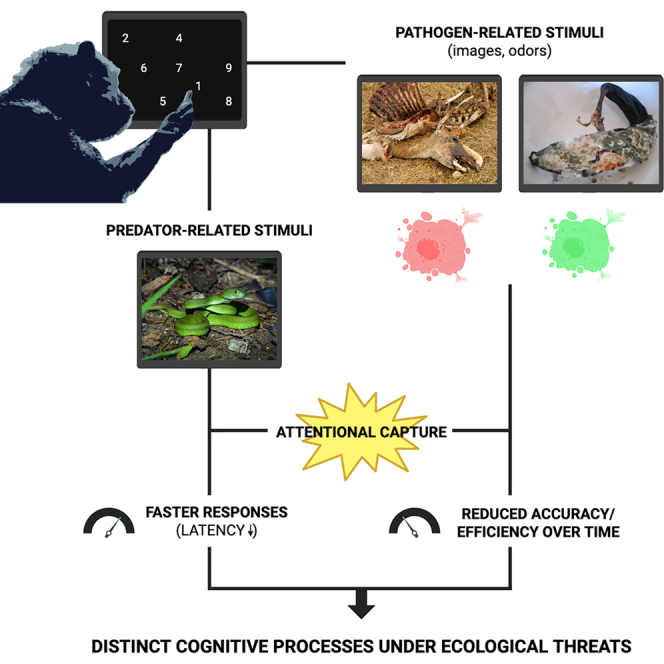

## Teaser

Natural sciences; Biological sciences; Zoology; Evolutionary biology

## Introduction

Disgust and fear are two emotions that evolved to serve different adaptive functions. While disgust primarily protects from disease by motivating the avoidance of pathogens, parasites, and toxins,^1^^,^^2^^,^^3^ fear facilitates escape from immediate threats, such as predators, via fight-or-flight responses.^4^^,^^5^^,^^6^^,^^7^^,^^8^ The differing consequences of these threats—from acute or chronic disease and reduced fitness in the case of infection to immediate death in the case of predation—may have led to the evolution of distinct responses. In humans, disgust is expressed by facial expressions that block the entry of potential toxins or pathogens (narrowed eyes, nose wrinkling, and raised upper lip),^9^^,^^10^^,^^11^ oculomotor avoidance after initial processing,^12^^,^^13^^,^^14^ and physiological reactions such as pupil constriction^15^^,^^16^—or dilation^17^—an inconsistency, which could be due to differences in stimulus type. Disgust also disrupts gastric rhythm,^18^^,^^19^ reduces heart rate,^20^^,^^21^ and activates the anterior insula.^22^^,^^23^ By contrast, fear manifests through expressions that enhance sensory perception (raised eyebrows, nose elongation, and stretched lips),^10^ early and sustained attention to the threat,^24^^,^^25^ along with pupil dilation,^17^^,^^26^ elevated heart rate,^27^ adrenaline release,^28^ and amygdala activation.^29^ Disparities in perception and outcomes may have contributed to the research gap between these two emotions, with fear historically receiving more attention, though interest in disgust has markedly increased since the 2000s.^30^^,^^31^ Disgust has been understudied not only in humans but across species.^32^ Moreover, these two emotions are frequently examined in isolation, even though threats from pathogens and predators often co-occur within the same environments.^31^^,^^33^ This siloed approach limits the ecological validity of current findings. Understanding how disgust and fear interact—or diverge—is crucial for uncovering the evolutionary mechanisms underlying threat detection and behavioral responses, particularly in our closest phylogenetic relatives, where emotional functioning remains largely unexplored.

Recent research suggests that non-human primates display core disgust responses similar to those observed in humans, including characteristic facial expressions,^34^^,^^35^^,^^36^ physiological reactions like nausea,^35^ anterior insula activation,^34^^,^^35^ and avoidance behaviors.^37^^,^^38^^,^^39^^,^^40^^,^^41^^,^^42^^,^^43^^,^^44^ Despite increasing evidence of emotional expressions and physiological responses associated with disgust in non-human primates, its cognitive dimension—how it modulates attention and decision-making—has received little empirical investigation. In contrast, fear has been extensively studied behaviorally, cognitively, and physiologically.^45^^,^^46^ Notably, both emotions have been primarily examined through visual stimuli in primates,^47^^,^^48^ while threat perception may involve sensory diversity.^49^^,^^50^^,^^51^

In this study, we explored the effect of pathogen-versus predator-related stimuli on cognitive processes in captive trichromatic chimpanzees (*Pan troglodytes*; Table S1), building on previous findings of chimpanzees’ avoidance behaviors toward pathogen-associated cues.^37^^,^^52^ We conducted four experiments to address the following questions: (1) Do pathogen-related images influence cognitive performance, here defined as the accuracy and latency with which individuals process and respond to task stimuli? (2) Do pathogen-associated odors influence cognitive performance? (3) Does cognitive performance differ under pathogen-versus predator-related stimuli? (4) Do attentional patterns differ between pathogen- and predator-related stimuli? To test these, chimpanzees completed a number ordering task,^53^ a standard touchscreen test of working memory and attention, while being presented with visual or olfactory stimuli. Attentional responses were measured using eye-tracking. In line with the hypothesis that disgust promotes the avoidance of physical and perceptual contact with pathogen sources,^54^^,^^55^^,^^56^ we predicted that pathogen-related stimuli would impair cognitive performance (e.g., reduce accuracy, reactivity and/or efficiency), while predator-related stimuli would enhance it due to heightened vigilance^57^ (Table 1). According to the eye-mind hypothesis—that gaze reflects attention and thought^59^—we also expected that pathogen-related images would divert gaze and reduce attentional focus, while predator images would sustain visual attention. Understanding how these emotions modulate cognition offers insight into their adaptive roles in guiding behavior under threat.

**Table 1:** Hypothesized and observed effects of pathogen- and predator-related cues on task performance and attention across dependent variables

Cue type	Hypothesis	Dependent variable	Prediction	Observation			
				Exp1	Exp2	Exp3	Exp4
Pathogen	disgust induces cognitive interference (Krusemark and Li^47^)	accuracy			X	X	NA
		reaction time		X	X	X	
		latency					
	disgust induces avoidance (Curtis et al.^54^; Armstrong et al.^14^)	attention capture		NA			
		sustained attention					X ()
Predator	fear optimizes cognitive processes (Krusemark and Li^47^; Van Le et al.^58^) and facilitates escape (Qi et al.^8^)	accuracy		NA		X	NA
		reaction time				X	
		latency					
	fear increases vigilance (tonic alertness, Krusemark and Li^47^; and sustained attention, Yorzinski et al.^25^)	attention capture				NA	
		sustained attention					

Predicted effect directions are indicated by arrows:  increase,  decrease. Observed effects reflect model-estimated effects of cue condition on each dependent variable. “✔” indicates support for the predicted effect, “X” indicates no support or an effect in the opposite direction (specified in parentheses), and “NA” indicates that the measure or cue type was not assessed in that experiment.

## Results

### Pathogen-related images elicit distinct performance patterns

**Figure 1:**
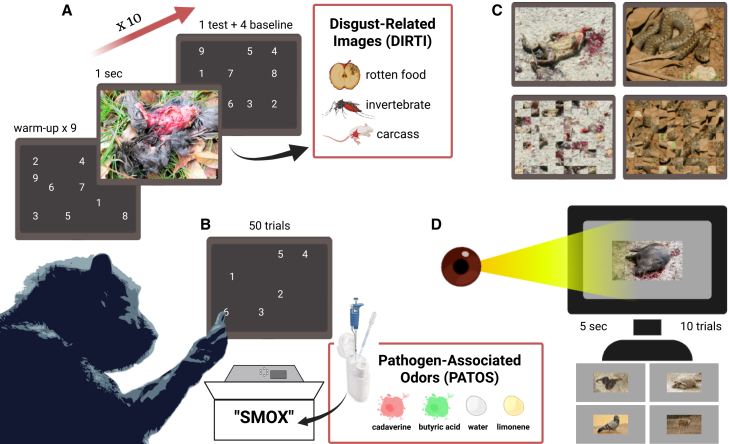
Experimental designs of number ordering and eye-tracking tasks (A) Experiment 1: chimpanzees performed a number ordering task while being repetitively exposed to disgust-related images (rotten food, disease-associated/pathogen-mimicking invertebrates, heterospecific carcasses) or matched control images presented briefly on touch screens. (B) Experiment 2: chimpanzees performed the number ordering task while exposed to pathogen-associated odors (cadaverine, butyric acid) or control odors diffused at low concentrations through a nebulizer connected to a custom odor box (SMOX). To reduce potential discomfort, trials involved six rather than nine numerals. (C) Experiment 3: new heterospecific animal carcass, snake or control scrambled images of those were presented at regular intervals on touch screens during the number ordering task using the same paradigm as in A. (D) Experiment 4: chimpanzees’ eye-tracking responses were recorded while viewing carcass, snake and control images. Figure created with BioRender. Original chimpanzee (Ai) artwork, Kenneth Keuk.

We began by testing whether and which “disgust-related images” (DIRTI,^60^ see supplemental information; Figures S1–S3) influence chimpanzee performance on a number ordering task—where numerals from 1 to 9 were presented simultaneously at random locations on a touchscreen and had to be selected in ascending order (Figure 1A). Every five trials, a full-screen image was displayed: either a pathogen-related one—such as a heterospecific animal carcass (mammal, bird, amphibian or marsupial, at various stages of decay), rotten food (spoiled fruits or vegetables), invertebrates associated with disease risk (e.g., tick, mosquito)—or a corresponding control image (a sleeping animal, fresh food, butterflies, or other non-pathogenic invertebrates). Given that the three control conditions did not significantly differ in the proportion of correct trials (*χ*^2^ (2) = 2.20, *p* = 0.333), they were combined into a single control category for subsequent analyses. Performance was measured via trial accuracy (correct vs. incorrect), reactivity (latency to touch the first numeral), and time to complete correct trials.

The generalized linear mixed model (GLMM) for accuracy (M1.1) including the interaction between condition and trial number did not significantly improve model fit over the additive model without the interaction (likelihood ratio test, LRT; Δ*LogLik* = 3.6, Δ*d*.*f*. = 3, *p* = 0.062), but the additive model outperformed the null model (Δ*LogLik* = 4.5, Δ*d*.*f*. = 3, *p* = 0.031, see Table S2). We therefore retained the additive model. The full model for reactivity (M1.2), however, did not outperform its null model (Δ*LogLik* = 5.1, Δ*d*.*f*. = 6, *p* = 0.114) and was therefore not retained. In contrast, for latency to complete correct trials (M1.3), the model including the interaction between condition and number of image exposures significantly outperformed both its null (Δ*LogLik* = 9.4, Δ*d*.*f*. = 6, *p* = 0.004) and additive (Δ*LogLik* = 7, Δ*d*.*f*. = 3, *p* = 0.003) models and was retained for interpreting effects of image condition.

**Figure 2:**
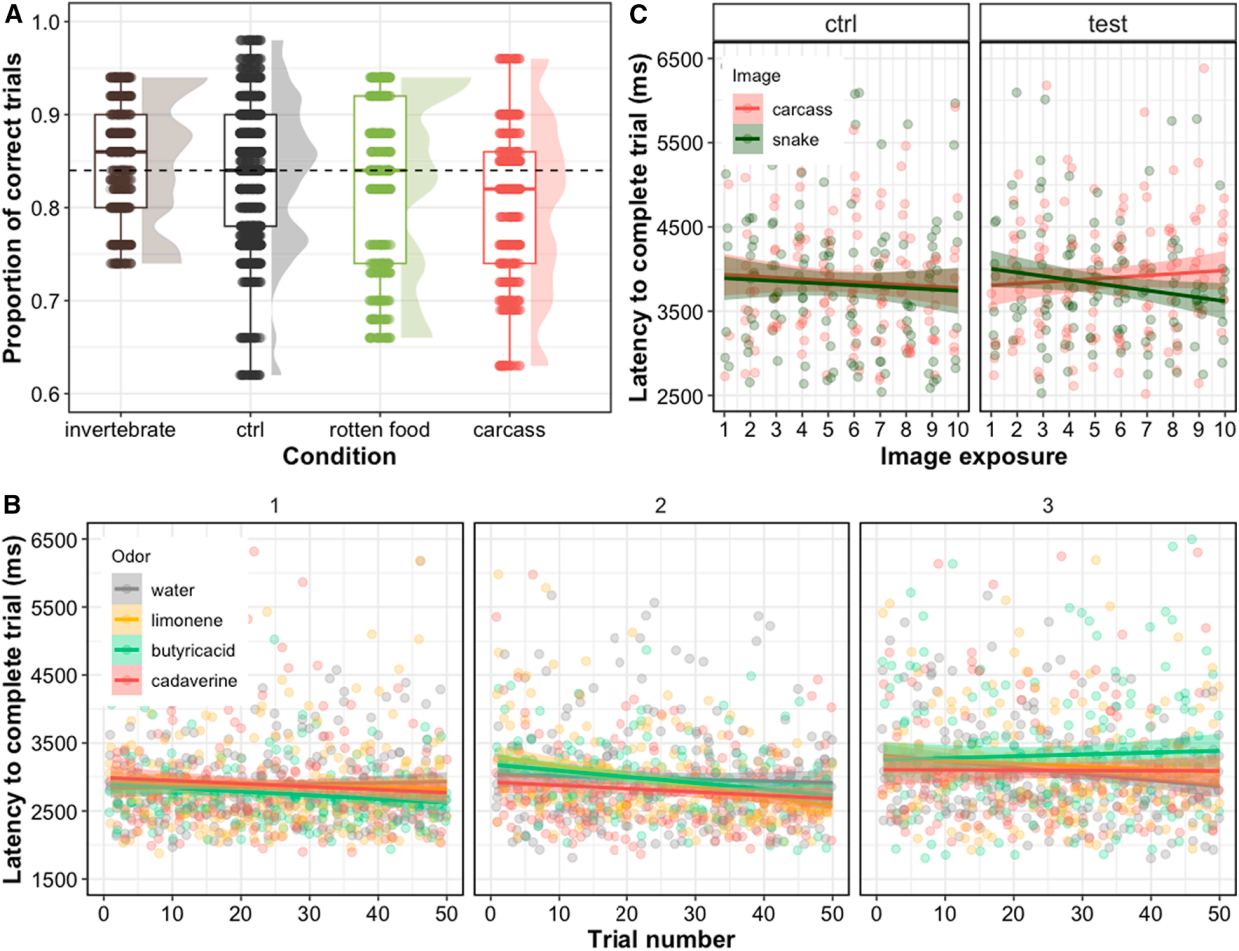
Effect of disgust- and fear-related stimuli on chimpanzee cognitive performance in the number ordering task (A) Experiment 1: raincloud plots show the proportion of correct trials (median and interquartile range) during sessions with images of pathogen-associated/mimicking invertebrates (brown), controls (black), rotten food (green), and animal carcasses (red). The dashed horizontal line indicates the average proportion of correct trials in the control condition (0.838). Chimpanzees made significantly more errors when exposed to carcass images compared to invertebrate images (GLMM; *p* < 0.05), but not compared to rotten food or control images. (B) Experiment 2: scatterplots display the latency to complete correct trials (in ms) under exposure to water (gray), limonene (yellow), butyric acid (green), and cadaverine (red) odors in sessions 1–3. Chimpanzees were significantly slower under repeated exposure to butyric acid than under water (GLMM; *p* < 0.05). (C) Experiment 3: scatterplots of latency (ms) to complete correct trials after exposure to carcass (red) and snake (green) images. Chimpanzees were significantly faster under repeated exposure to snake images compared to carcass images (GLMM; *p* < 0.01).

The proportion of correct trials was high across all conditions (invertebrate: 0.85, control: 0.84, rotten food: 0.83, carcass: 0.80; Figure 2A). However, the GLMM revealed significant differences in accuracy across image conditions (*χ*^2^ (3) = 8.9, *p* = 0.031; Tables 2 and S2). Accuracy was significantly lower in the carcass condition compared to the control (*β* = −0.24 ± 0.10 SE, *z* = −2.44, *p* = 0.015), while differences for the invertebrate (*β* = 0.11 ± 0.11 SE, *z* = 0.96, *p* = 0.335) and rotten food (*β* = −0.10 ± 0.10 SE, *z* = −0.93, *p* = 0.354) conditions were not significant (Table S3). Trial number itself did not influence accuracy (*β* = 0.04 ± 0.04 SE, *z* = 0.99, *p* = 0.323). The estimated marginal means confirmed this pattern: invertebrate = 1.80 [1.44, 2.16], control = 1.69 [95% confidence interval, CI: 1.37, 2.02], rotten food = 1.60 [1.24, 1.95], and carcass = 1.45 [1.10, 1.80]. Post hoc pairwise comparisons indicated that the effect was mainly driven by the difference between carcass and invertebrate conditions (*β* = −0.35 ± 0.13 SE, z = −2.74, *p* = 0.031; Table S4).

**Table 2:** Summary of significant effects across experiments, stimuli, and dependent variables

Experiment	Stimulus contrast	Dependent variable	Effect
1	carcass vs. invertebrate	accuracy	**↓**
	rotten food vs. carcass/control/invertebrate	latency	**↑**
2	butyric acid vs. water	latency	**↓↑**
	cadaverine vs. water	latency	**↓**
	limonene vs. water	latency	**↑**
3	carcass vs. snake	latency	**↑**
4	carcass vs. pigeon	first fixation	**↑**
	snake vs. pigeon	first fixation	**↑**
	carcass vs. deer	proportion of fixations	**↑**
	carcass vs. pigeon	proportion of fixations	**↑**
	snake vs. deer	proportion of fixations	**↑**
	snake vs. pigeon	proportion of fixations	**↑**
	deer vs. tortoise	proportion of fixations	**↓**
	carcass vs. deer	proportion of time looking	**↑**
	carcass vs. pigeon	proportion of time looking	**↑**
	snake vs. deer	proportion of time looking	**↑**
	snake vs. pigeon	proportion of time looking	**↑**
	snake vs. tortoise	proportion of time looking	**↑**
	deer vs. tortoise	proportion of time looking	**↓**

Arrows indicate the direction of the effect relative to the comparison condition (↑ higher value; ↓ lower value). For experiment 2, “↓↑” indicates a change in direction across sessions. Only significant contrasts are shown; full statistical results are reported in Tables S4, S6, S8, and S10.

Task efficiency, measured by the latency to complete trials, significantly improved with exposure to rotten food images compared to the control (*β* = −0.04 ± 0.01 SE, *z* = −3.13, *p* = 0.002; Table S3), whereas other conditions did not differ from the control (invertebrate: *β* = 0.00 ± 0.01 SE, *z* = 0.23, *p* = 0.816; carcass: *β* = 0.02 ± 0.01 SE, *z* = 1.16, *p* = 0.247). Post hoc pairwise comparisons showed that chimpanzees were slower after being exposed to the first few rotten food images (versus control: *β* = 0.10 ± 0.03 SE, *z* = 3.34, *p* = 0.005; versus invertebrate: *β* = 0.12 ± 0.03 SE, *z* = 3.53, *p* = 0.002; versus carcass: *β* = 0.13 ± 0.03 SE, *z* = 3.69, *p* = 0.001; Table 2), but that effect did not persist after multiple exposures (see Table S4).

### Pathogen-associated odors elicit distinct performance patterns over time

Building on the effects of pathogen-related images, we next tested whether pathogen-associated odors similarly affect cognitive performance. In this second experiment, we used the same paradigm as before but replaced the visual stimuli with olfactory ones. We diffused low concentrations (see STAR Methods) of different odors—cadaverine (associated with putrefaction), butyric acid (vomit-like), limonene (citrus) or water—through a box and nebulizer built just beneath the touchscreen, while chimpanzees performed the task (Figure 1B; Video S1). To shorten the sessions and minimize potential discomfort, the number of numerals presented on the screen was reduced from nine to six.

Video S1. Chimpanzee Ai performing the number ordering task under the cadaverine condition (session 1, experiment 2) Low concentrations of cadaverine were released beneath the screen while chimpanzees completed the task.

Only the GLMM for latency to complete correct trials (M2.3) showed a significantly better fit when including the interactions between odor, trial, and session numbers compared to both the null (LRT; Δ*LogLik* = 16, Δ*d*.*f*. = 13, *p* = 0.001) and the additive (without interactions; Δ*LogLik* = 15, Δ*d*.*f*. = 10, *p* < 0.001) models (Table S2). We therefore retained this interaction model for interpretation.

Overall, latency to complete the task increased by approximately 5%–6% across trials and sessions under both cadaverine (GLMM; *β* = 0.06 ± 0.02 SE, *z* = 3.60, *p* < 0.001) and butyric acid (*β* = 0.05 ± 0.02 SE, *z* = 3.12, *p* = 0.001) relative to the control water condition, whereas the effect of limonene was not significant (*β* = 0.02 ± 0.02 SE, *z* = 1.40, *p* = 0.160; Figure 2B; Table S5). Post hoc pairwise comparisons revealed more complex temporal dynamics. During the last third of the first session (after ≈4 min of diffusion), chimpanzees were faster under cadaverine (*β* = −0.12 ± 0.05 SE, *z* = −2.57, *p* < 0.05) and butyric acid (*β* = −0.14 ± 0.05 SE, *z* = −2.75, *p* = 0.031) compared to water (Tables 2 and S6). This effect reversed during the first two-thirds of the second session, though differences were not significant. In the middle to late portion of the third session, butyric acid slowed latency relative to water (mid: *β* = 0.11 ± 0.04 SE, *z* = 3.04, *p* = 0.013; high: *β* = 0.16 ± 0.05 SE, *z* = 3.12, *p* < 0.01), whereas cadaverine showed a similar but non-significant trend (mid: *β* = 0.04 ± 0.04 SE, *z* = 1.07, *p* = 0.708; high: *β* = 0.12 ± 0.05 SE, *z* = 2.29, *p* = 0.102). Although limonene did not affect overall latency, chimpanzees responded more slowly in early trials of the second session compared to the water condition (*β* = 0.14 ± 0.05 SE, *z* = 2.70, *p* = 0.035), consistent with the direction of the effects observed for cadaverine and butyric acid.

### Carcass and snake images have opposite effects on task efficiency over time

In a third experiment, we used new sets of carcass images—identified in experiment 1 as eliciting the lowest proportion of correct trials—and compared their effect to snake images, using the same paradigm. We used scrambled versions of the carcass and snake images as controls (Figure 1C).

Only the GLMM for latency to complete correct trials (M3.3) with interactions between condition, image type, and number of exposures outperformed its respective null (LRT; Δ*LogLik* = 7.1, Δ*d*.*f*. = 6, *p* = 0.027) and additive (without the interaction; Δ*LogLik* = 6.9, Δ*d*.*f*. = 4, *p* = 0.008) models (Table S2).

Chimpanzees completed the task significantly faster when exposed to snake images compared to carcass (GLMM; *β* = −0.06 ± 0.02 SE, *z* = −2.59, *p* < 0.001) images (Figure 2C; Table S7; Videos S2 and S3). Pairwise post hoc comparisons confirmed this effect, showing that repeated exposure to snake images improved performance by 10% compared to carcass images (*β* = −0.10 ± 0.03 SE, *z* = −3.17, *p* = 0.006; Table 2). Other comparisons at minimum, mid, and high image exposure were not significant (all *p* > 0.090; see Table S8).

Video S2. Chimpanzee Cleo performing the number ordering task under the snake condition (session 2, experiment 3) Snake images were displayed full screen every five trials over 50 trials. Chimpanzees performed faster in this condition compared to the carcass condition.

Video S3. Chimpanzee Cleo performing the number ordering task under the carcass condition (session 1, experiment 3) Images of heterospecific animal carcasses were displayed full screen every five trials over 50 trials. Chimpanzees performed slower in this condition compared to the snake condition.

### Carcass and snake images attract and retain attention

In the fourth experiment, we used an eye-tracking task to present chimpanzees with new sets of carcass and snake images, as well as control images of tortoises, pigeons, and deer. This allowed us to measure their visual attention and test whether attentional patterns could explain the performance differences observed in experiment 3. We used three proxies for visual attention: the location of the first fixation (on the animal or not), the proportion of fixations on the animal versus elsewhere (i.e., the environment in the picture or the gray area surrounding it; Figure 1D; see Figure S4) and the proportion of time spent looking at the animal versus looking elsewhere (hereafter, attention score).

All three GLMMs (M4.1-3) with their main effect were significantly better than their null, intercept-only, models (LRT; first fixation location: Δ*LogLik* = 14.9, Δ*d*.*f*. = 4, *p* < 0.001; proportion of fixations: Δ*LogLik* = 32.7, Δ*d*.*f*. = 4, *p* < 0.001; proportion of time looking: Δ*LogLik* = 42.7, Δ*d*.*f*. = 4, *p* < 0.001; Table S2).

#### First fixation location

**Figure 3:**
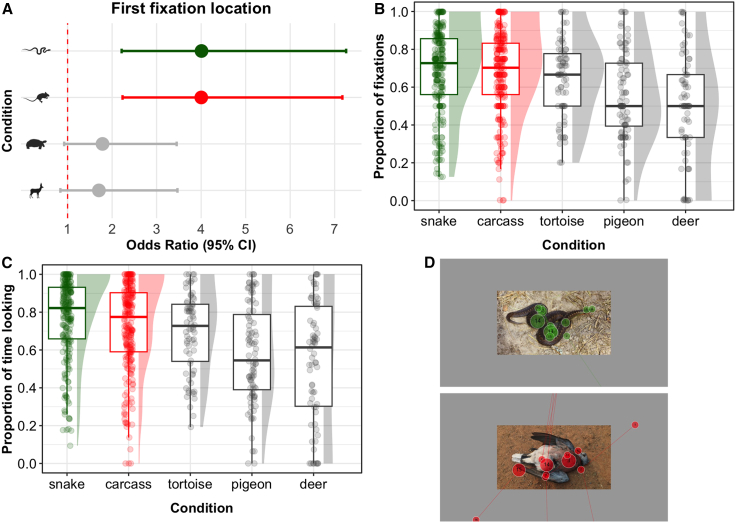
Effect of disgust- and fear-related images on patterns of visual attention in experiment 4 (A) Forest plots show the odds ratios of the first fixation location on snakes (green), carcasses (red) and control animals (gray), taking the pigeon images as referential. Chimpanzees’ first fixation had significantly more chances to land on the snake or carcass than on the pigeon (GLMM; *p* < 0.001). (B) Raincloud plots for the proportion of fixations (median and interquartile range) on snake (green), carcass (red) and control animal (gray) images. Chimpanzees had more fixations on snakes and carcasses compared to deer and pigeons (GLMM; all *p* < 0.001). (C) Raincloud plots for the proportion of time looking (median and interquartile range) at the snake, carcass and control animal in the image vs. looking away (i.e., attention score). Chimpanzees looked at carcasses and snakes for longer than at control animals (GLMM; all *p* < 0.01). However, there was no significant differences between snakes and carcasses (*p* = 0.210). (D) Gaze plots of chimpanzees looking at a snake (top, Ayumu) and a bird carcass (bottom, Ai). Fixations are shown as circles, with size proportional to fixation duration. Lines between circles indicate saccades—rapid eye movements during which vision is suppressed.

Chimpanzees’ first fixation was significantly more likely to land on the animal in carcass images compared to control images (GLMM; all *p* < 0.01; Figure 3A; Table S9). However, there was no significant difference in the likelihood of first fixating on the animal in carcass versus snake images (*β* < −0.01 ± 0.24 SE, *z* = −0.01, *p* = 0.995). Pairwise post hoc comparisons indicated that this effect was mainly driven by contrasts with pigeon images (carcass–pigeon: *β* = 1.39 ± 0.30 SE, *z* = 4.66, *p* < 0.001; snake-pigeon: *β* = 1.39 ± 0.30 SE, *z* = 4.58, *p* < 0.001; Table 2), whereas all other comparisons were non-significant (*p* > 0.06; Table S10).

#### Proportion of fixations

Chimpanzees directed a significantly greater proportion of fixations toward the animal in carcass images than in control images (GLMM; all *p* < 0.05, Figure 3B; Table S9), whereas no difference was found relative to snake images (*β* = −0.03 ± 0.06 SE, *z* = −0.53, *p* = 0.599). Pairwise post hoc comparisons revealed that animals in carcass images received more fixations than those in deer (*β* = 0.66 ± 0.10 SE, *z* = 6.90, *p* < 0.001) and pigeon (*β* = 0.45 ± 0.09 SE, *z* = 5.05, *p* < 0.001) images, but not more than in snake (*β* = −0.03 ± 0.06 SE, *z* = −0.53, *p* = 1.000) or tortoise (*β* = 0.19 ± 0.10 SE, *z* = 2.01, *p* = 0.260; Tables 2 and S10) images. Similarly, animals in snake images attracted more fixations than those in deer (*β* = 0.69 ± 0.10 SE, *z* = 7.18, *p* < 0.001) and pigeon (*β* = 0.49 ± 0.09 SE, *z* = 5.31, *p* < 0.001) images, but not more than in tortoise images (*β* = 0.23 ± 0.10 SE, *z* = 2.31, *p* = 0.142), which in turn received more fixations than deer images (*β* = 0.47 ± 0.11 SE, *z* = 4.28, *p* < 0.001).

#### Attention score

Overall, chimpanzees spent significantly more time looking at snakes than at carcasses (GLMM; *β* = 1.16 ± 0.07 SE, *z* = 2.12, *p* = 0.034; Figures 3C and 3D; Table S9). In contrast, the proportion of time spent looking at the animals (versus elsewhere) was significantly lower in control images than in carcass images (tortoise: *β* = −0.25 ± 0.12 SE, *z* = −2.15, *p* = 0.032; pigeon: *β* = −0.57 ± 0.11 SE, *z* = −5.25, *p* < 0.001; deer: *β* = −0.79 ± 0.12 SE, *z* = −6.83, *p* < 0.001). Pairwise post hoc comparisons showed that both carcasses and snakes elicited longer total looking times than deer and pigeons (carcass–deer: *β* = 0.79 ± 0.12 SE, *z* = 6.83, *p* < 0.001; carcass-pigeon: *β* = 0.57 ± 0.11 SE, *z* = 5.25, *p* < 0.001; snake-deer: *β* = 0.94 ± 0.12 SE, *z* = 8.08, *p* < 0.001; snake-pigeon: *β* = 0.72 ± 0.11 SE, *z* = 6.55, *p* < 0.001; Table 2). Chimpanzees looked at snakes for longer than tortoises (*β* = 0.40 ± 0.12 SE, *z* = 3.44, *p* = 0.005), which in turn received longer looking times than deer (*β* = 0.54 ± 0.13 SE, *z* = 4.10, *p* < 0.001). However, the difference in total looking time between carcasses and snakes was not significant in the post hoc tests (*β* = −0.16 ± 0.07 SE, *z* = −2.12, *p* = 0.210; Table S10), suggesting that the main effect detected by the GLMM was influenced by contrasts among several control conditions.

## Discussion

Taken together, our results suggest that both pathogen-related and predator-related stimuli capture and sustain attention in chimpanzees, but differ in their effects on cognitive performance: pathogen-related cues tended to interfere with task performance, whereas predator-relevant stimuli such as snake images were associated with improved performance. These findings support the hypothesis that fear of predators facilitates escape (or fight) by optimizing cognition and maintaining attention,^5^^,^^6^^,^^8^ whereas disgust toward pathogen cues promotes avoidance through initial attention and subsequent cognitive modulation.^47^^,^^61^^,^^62^^,^^63^

Among the pathogen-related images of experiment 1, heterospecific animal carcasses were associated with lower accuracy compared to disease-associated invertebrates. When considering task efficiency (latency), chimpanzees completed trials more slowly under rotten food images, particularly at the beginning of a session. Over time, however, latencies decreased, suggesting a possible habituation or learning effect specific to the rotten food stimuli. In the other conditions, their latency remained relatively stable over time.

These contrasting effects suggest that pathogen-related stimuli may not operate as a homogeneous category but instead engage partially distinct functional subtypes. Carcasses, rotten food, and disease-associated invertebrates may signal different routes of disease transmission and varying levels of pathogen or toxin risk, potentially recruiting different avoidance strategies. For instance, carcasses may represent highly salient cues of death and infection and are among the strongest human disgust elicitors,^60^^,^^64^ whereas rotten food may primarily signal intoxication risk. The absence of strong effects for invertebrate images suggests that not all pathogen-relevant or mimicking cues engage the same defensive processes, possibly reflecting different underlying systems (e.g., gut vs. skin-related defenses).^65^^,^^66^ While our statistical models accounted for subject variation, we observed striking behavioral responses in one female (Pendesa), who turned her back to the screen or knocked on the window when viewing disease-associated invertebrates (see Video S4), suggesting an influence of subject experience. In humans, disgust is structured into partially dissociable domains (e.g., core, animal-reminder, contamination-related)^67^^,^^68^^,^^69^; our findings raise the possibility that similar functional differentiation may exist in chimpanzees. While further work is needed to test this explicitly, these unpredicted results provide preliminary evidence that disgust in non-human primates may not constitute a unitary system.

Video S4. Chimpanzee Pendesa performing the number ordering task under the disease-associated invertebrate condition (session 2, experiment 1) Images of invertebrates were displayed full screen every five trials, following nine initial warm-up trials. Pendesa’s number set was reduced from 9 to 6 to accommodate her motivation and capacities. Here, she turns away from the screen when presented with picture 3.

Pathogen-associated odors, such as cadaverine and butyric acid, showed dynamic effects on task performance across sessions. During the first exposure, both odors were associated with shorter latencies to complete trials relative to water, suggesting a transient facilitation of performance that may reflect heightened arousal or an attempt to reduce exposure to the aversive stimulus. However, this effect did not persist: by the third session, butyric acid in particular was linked to significantly longer latencies, indicating reduced efficiency. Interestingly, a transient slowing also occurred early in the second session with limonene, a salient non-pathogen-related odor, suggesting that olfactory interference rather than aversion alone can momentarily affect performance. Taken together, these temporal shifts suggest that pathogen-related cues may initially elicit an alert or avoidance-oriented response, but later promote withdrawal or cognitive interference upon repeated exposure.^14^^,^^63^^,^^70^ However, because these interpretations rely primarily on latency measures, the underlying mechanisms (e.g., arousal, avoidance, or cognitive load) cannot be distinguished without complementary physiological indices (e.g., heart rate, skin conductance, nasal temperature). While cadaverine could theoretically indicate disease and predation risk, the overall pattern is more consistent with disgust than fear responses. In line with human findings showing that brief, high-concentration exposure to putrescine elicits faster reaction times (interpreted as heightened vigilance),^70^ our lower concentrations and longer exposure revealed a later slowing and more cautious responding, reflecting the temporal dynamics of risk processing.

Both substances appear to elicit innate aversion in human neonates and fish,^71^^,^^72^ possibly due to the risks they signal: intoxication (butyric acid) and death (cadaverine). That cadaverine affected the behavior of captive chimpanzees—that may or may not have prior exposure to decaying flesh—supports the interpretation of an evolved, rather than learned, response. Cadaverine and putrescine are two of the main volatile organic compounds released during the decomposition of flesh. Prior studies have shown that these compounds are aversive to non-scavenging species, including humans,^70^ chimpanzees,^52^ and zebrafish (*Danio rerio*),^72^ and can induce burial behavior in rats (*Rattus norvegicus*).^73^ In chimpanzees, this translates by spending less time near an object with putrescine compared to ammonia or water, although at much higher concentrations (3.5 g on cotton pads) than used here. Despite our lower concentration (0.3% for both cadaverine and butyric acid), we still observed some participation refusals (11% of experimental days), primarily for these two odors. We found no significant differences in performance between cadaverine and butyric acid, suggesting that the two odors may have been processed similarly in the context of the task, possibly reflecting comparable perceived intensity or valence and/or belonging to the same functional category.

Repeated exposure to carcass images significantly increased the latency to complete trials compared to snake images. These contrasting trends were not significant relative to their respective controls but suggest distinct temporal dynamics across stimulus types. Initially, chimpanzees responded faster under carcass images and slower under snake images compared to controls. However, after the fourth exposure (Figure 2C), a divergence emerged: latencies for carcass images continued to increase, whereas latencies for snake images steadily decreased. This pattern suggests a differential effect of repeated exposure. The first exposure to snake images may have induced heightened alertness or caution, temporarily slowing responses. Over repeated exposures, this heightened state could have enhanced cognitive function—leading to faster reaction times, improved focus, or more effective decision-making—as chimpanzees became better at processing ecologically relevant threats. An alternative explanation is habituation, but this seems less likely since different snake species and images were presented across trials and sessions, reducing stimulus familiarity. Moreover, the chimpanzees were highly experienced with the number-ordering task and the testing procedure, making increased task familiarity an unlikely explanation for the observed temporal changes. This differs from the improved performance seen with repeated exposure to rotten food images, where greater familiarity and prior experience may facilitate habituation or learning effects.

The divergent patterns observed—faster responses to repeated predator-related stimuli (snakes) and slower responses to pathogen-related stimuli (carcasses)—mirror findings in humans.^10^^,^^74^^,^^75^ For instance, Krusemark and Li^47^ showed that fear-inducing images (snakes, spiders, but see Polák et al.,^76^ and guns) decrease reaction times, while disgust-inducing images (cockroaches, feces, and vomit) increase them in a visual search task. This contrast supports the interpretation that fear can facilitate vigilance and rapid information processing, whereas disgust may induce avoidance or cognitive interference. However, these behavioral differences do not necessarily imply entirely separate mechanisms. Instead, they likely arise from interactions among perceptual, attentional, and motivational systems rather than discrete emotional modules, with fear and disgust potentially modulating cognition through partially overlapping but temporally distinct processes.^77^^,^^78^ Our data thus parallel human evidence and could be consistent with a shared evolutionary architecture for emotion-cognition interactions in chimpanzees. Furthermore, evidence of snake-sensitive neurons deep in the visual system of non-human primates^58^ supports the idea of an evolved mechanism for rapid detection and efficient processing of threatening stimuli. In line with this, recent experiments showed that chimpanzees fixated more on the eyes of forward-facing lions than on those of averted lions or impalas, even without prior exposure to these species, pointing to an evolved rather than learned sensitivity to predator gaze.^79^

Snake and carcass images both captured visual attention more effectively than control images, as indicated by first fixation location and the number (proportion) of fixations—consistent with previous findings showing faster detection of threatening versus non-threatening stimuli in humans^80^^,^^81^^,^^82^ and macaques.^83^^,^^84^ This pattern was somewhat contrary to our prediction that pathogen-related stimuli would divert gaze and reduce attentional focus. On average, chimpanzees allocated about 71% of their viewing time to snakes and 67% to carcasses, compared to 62% for tortoises, 54% for pigeons, and 49% for deer. This pattern indicates that both fear- and disgust-relevant cues strongly maintained visual attention. Although the difference between snakes and carcasses was not significant, snakes tended to elicit slightly more sustained attention, possibly because snakes represent an active threat that could approach and attack, whereas carcasses are inert.^85^ Indeed, features displayed in the carcass images (e.g., visible bones, smashed body, viscera) may have signaled to the chimpanzees that the carcass would not move. Previous eye-tracking studies have also shown that chimpanzees pay more attention (i.e., higher fixation durations) to death-related stimuli, such as conspecific skulls, compared to other species or control stimuli.^86^

Eye-tracking studies in humans show that disgusting images initially attract gaze (for about the first 2 s or the first trial) and subsequently trigger attentional avoidance,^13^^,^^14^^,^^63^ likely reflecting an evolved tendency to first assess threat and then avoid potential contamination. To clarify the temporal dynamics of chimpanzees’ attention to carcass images, future analyses should examine gaze allocation across time. In contrast, snake images are known to both capture and hold visual attention in human^25^^,^^87^ and non-human primates,^88^ (though see Zeller et al.),^89^ which aligns with the hypothesis that snakes have been a key evolutionary threat for primates.^90^^,^^91^ Taken together, these results suggest that attentional capture does not uniformly translate into improved task performance. While snake images may sustain attention in ways that facilitate vigilance and task execution, carcass images may capture attention in a manner that interferes with ongoing cognitive processing. Because attentional allocation and task performance were assessed in separate paradigms and involved different stimulus presentation durations, this relationship should nonetheless be interpreted cautiously.

Interestingly, we also observed significant differences across the control conditions, with tortoises receiving more fixations and being looked at longer than deer. While not central to our hypotheses, these differences are informative: reptiles such as tortoises may attract more visual attention due to their distinctive morphology (e.g., the shell), which is evolutionarily more distant from primates and therefore may appear more novel or ambiguous with respect to potential threat. Elsewhere, Testudines have been used as control stimuli to test the snake detection theory using diverse methodologies such as immersive virtual reality, attentional interference tasks, and emotional/physiological ratings, with findings consistently showing that compared to Testudines, snakes elicit stronger avoidance behaviors, greater attentional capture, and more negative affective and physiological responses.^92^^,^^93^^,^^94^ However, other research suggests that features shared with snakes, such as reptilian scale patterns, may themselves trigger enhanced visual attention, raising the possibility that tortoises could partially activate evolved threat-detection mechanisms due to their morphological similarity.^95^

Given the relatively small number of subjects involved in our study, inherent to great ape cognition research, we cannot rule out the influence of individual histories, temperament or prior experiences with ecologically relevant stimuli on sensitivity to threat-related stimuli. All subjects except Ai were born in captivity, and Ai arrived at the Kyoto University Primate Research Institute (now the Center for the Evolutionary Origins of Human Behavior) at one year old. Nevertheless, individuals may have had prior encounters with similar stimuli that could have shaped their responses during the experiments. For example, Pendesa displayed marked reactions to disease-associated invertebrate images, Chloe is known to be selective about seating on the booth floor if it has not been thoroughly cleaned beforehand, and Ayumu often displays inside experimental booths. Although these observations are anecdotal, they illustrate how individual tendencies related to contamination sensitivity or threat perception could contribute to variation in behavioral responses across subjects. Future work with larger samples or longitudinal designs would help clarify how individual experience and temperament modulate these responses.

### Limitations of the study

This study extends previous research by investigating cognitive processes under pathogen- and predator-related stimuli in a non-human primate. Our findings are therefore bounded in scope. For example, the fear-related effects reported here were elicited by snake images; it remains to be tested whether similar responses would emerge for other predator cues.^96^ Likewise, although we included pathogen-related odors, logistical constraints precluded the use of predator odors (e.g., felid urine; see Weiss et al.^97^). Incorporating these cues in future work would extend the comparative framework across sensory modalities. In addition, the number ordering task used here assessed immediate performance. Future studies using delayed or retention-based paradigms (e.g., delayed matching-to-sample task) could help determine whether threat-related cues influence longer term memory or learning processes. Finally, despite a relatively large and diverse stimulus set (90 images) in the eye-tracking experiment, luminance and contrast were not standardized across images, which limited our ability to analyze pupil size.

Our sample size was necessarily modest due to the practical constraints of testing chimpanzees trained to complete cognitive and eye-tracking tasks. Given the modest sample size, statistical power was low, increasing the likelihood of type 2 errors and inflating effect size estimates for significant findings. This is reflected in the small but significant latency changes observed in experiments 2 (+5%–6%) and 3 (−10%). Although within-subject designs increase statistical power, replication across additional sites with similarly trained individuals would strengthen confidence in the robustness of these findings.

More generally, incorporating complementary physiological measures alongside behavioral and eye-tracking data—such as heart rate variability, gastric activity or other autonomic indices—would help clarify whether the observed effects reflect disgust- or fear-related responses. However, collecting such measures in chimpanzees performing cognitive tasks would likely require additional instrumentation involving physical contact,^19^^,^^96^^,^^98^ which could interfere with task engagement. More broadly, although our findings are interpreted within an evolutionary framework, the link between the observed cognitive processes and fitness-relevant outcomes remains indirect. Longitudinal or ecologically embedded studies will be necessary to determine how such responses influence behavior and survival in naturalistic contexts.

### Conclusion

Our study provides evidence of how chimpanzees process pathogen- and predator-related stimuli in cognitive and eye-tracking tasks, revealing distinct patterns of facilitation and disruption in performance, alongside heightened attention compared to controls. These findings highlight the perceptual salience of ecologically relevant threats and point to promising avenues for future research. For instance, combining pathogen- and predator-related cues, whether within or across sensory modalities, could reveal how multiple threats jointly shape risk perception and behavior. Complementary tools such as ChimpFACS^99^ may help to identify specific facial muscle movements associated with exposure to carcass or snake stimuli. Further, comparing responses to live versus dead snakes would clarify whether animacy strengthens fear responses. Finally, examining how behavioral responses to potential threats spread socially—for example, whether observing conspecifics’ reactions influences an individual’s own avoidance or vigilance—will be essential to understanding the broader dynamics of threat perception.

## Resource availability

### Lead contact

Requests for further information and resources should be directed to and will be fulfilled by the lead contact, Cécile Sarabian (cecile.sarabian@swansea.ac.uk).

### Materials availability

This study did not generate new, unique reagents.

### Data and code availability

• Data reported in this paper are publicly available at GitHub (https://github.com/CSarabian/chimp-dead-vs-death.git).
• Code used to analyze the data are publicly available at GitHub (https://github.com/CSarabian/chimp-dead-vs-death.git).
• Any additional information required to reanalyse the data reported in this paper are available from the lead contact upon request.

## Acknowledgments

We thank the chimpanzee caretakers Akemi Hirakuri, Etsuko Ichino, and Tomoko Takashima for their help with the experiments. C.S. would like to thank Yena Kim, Kenneth Keuk, and Sylvain Dorrière for insightful discussions regarding data analyses and eye-tracking. C.S. would also like to thank Frans de Waal for stopping by her poster at IPS 2018 in Chicago and inspiring the exploration of the cognitive facet of disgust in non-human primates. We additionally thank three anonymous reviewers for their constructive comments, which helped improve the manuscript. Funding. This work was supported by the 10.13039/501100001691Japan Society for the Promotion of Science (postdoctoral fellowship P19088 to C.S.). C.S. also acknowledges funding from the Institute for Advanced Study in Toulouse (IAST) through the 10.13039/501100001665French National Research Agency (ANR) under grant ANR-17-EURE-0010 (program d’investissements d’avenir), and from a Marie Sklodowska-Curie postdoctoral fellowship funded by 10.13039/100014013UK Research and Innovation (UKRI) under the UK government’s 10.13039/100018693Horizon Europe funding guarantee, grant number EP/Z001625/1.

## Author contributions

Conceptualization, C.S.; methodology, C.S., I.A., and A.J.J.M.; investigation, C.S., A.G., and I.A.; writing – original draft, C.S.; writing – review and editing, C.S., A.J.J.M., N.K., A.G., and I.A.; funding acquisition, C.S.; resources, I.A. and A.J.J.M.; supervision, I.A., A.J.J.M. and N.K.

## Declaration of interests

The authors declare no conflicts of interest.

## Declaration of generative AI and AI-assisted technologies in the writing process

During the preparation of this work, the authors used ChatGPT to assist with language editing, specifically to shorten and improve the clarity of some sentences. After using this tool, the authors reviewed and edited the content as needed and take full responsibility for the content of the publication.

## STAR★Methods

### Key resources table

**Table:** 

REAGENT or RESOURCE	SOURCE	IDENTIFIER
**Deposited data**		

Analyzed data and code	Zenodo	https://doi.org/10.5281/zenodo.21154473

**Experimental models: Organisms/strains**		

Chimpanzees (*Pan troglodytes* spp.)	Center for the Evolutionary Origins of Human Behavior, Japan	N/A

**Software and algorithms**		

R	R Foundation	https://www.r-project.org/
Tobii Pro Lab v.1.181.37603	Tobii AB	https://www.tobii.com/
BioRender	Science Suite Inc.	https://www.biorender.com/

### Experimental model and study participant details

We conducted four experiments with nine adult chimpanzees (*Pan troglodytes* spp.; 6 females and 3 males; 35.7 ± 12.7 y.o.; see supplemental information, Table S1) at the Center for the Evolutionary Origins of Human Behavior (EHUB; formerly named Primate Research Institute) of Kyoto University, Inuyama campus, Japan between October 2020 and January 2022, with an amendment in August 2023 to include session 3 of the carcass condition, which had not been conducted previously. Chimpanzees were socially housed in two groups (A and G) in two indoor enclosures. Group A had continuous access to the enriched “green cage”, whereas the enriched “outdoor area” was used alternately by the two groups. They received a nutritionally balanced diet composed of fruits and vegetables, supplemented with nuts, had *ad libitum* access to water, and were provided with routine veterinary care. Participation in all experimental sessions was voluntary – by calling individual names to join a given laboratory (three at the time of the study) – and individuals were free to stop the experiments at any time. Not all subjects participated in all four experiments, as inclusion depended on task suitability. For the number ordering task, chimpanzees were included only after achieving ≥ 90% accuracy in three consecutive training sessions. In addition, one participant (Pendesa) was excluded from the eye-tracking experiment because an arachnoid cyst in her brain appeared to have partially affected her visual field (see^100^).

#### Ethics

The experimental protocol was approved by the Ethics Committee of the Primate Research Institute, Kyoto University (Approval #2019-212). All testing was conducted in dedicated indoor experimental booths (approx. 8 m^3^) located in the institute’s basement. Chimpanzees were called from their enriched outdoor enclosures and could freely choose whether to participate. If subjects stopped participating, the session was repeated when next possible. Food rewards were provided after each correct trial (experiments 1–3), and juice was offered ad-libitum during eye-tracking sessions (experiment 4). Each subject participated in brief sessions lasting 10–15 minutes, held in the morning or afternoon, and was not tested more than once per day under the same condition, but could be tested once in the morning and once in the afternoon under different conditions. The experimenters wore protective clothing and masks during tests to protect chimpanzees and themselves from potential exchange of pathogens. Experimental booths were cleaned after each subject experimental session.

### Method details

#### Apparatuses

Experiments 1–3 involved a number ordering task that chimpanzees at EHUB have performed for more than two decades.^53^^,^^101^^,^^102^^,^^103^ Nine numerals (one to nine) except for one subject (Pendesa: one to six) were presented at random locations on a 17” LCD touch screen (1280 × 1024 px) during one given trial. Chimpanzees had to touch the numerals in an ascending order. If correct, they received a small piece of apple or a dry raisin from a feeder connected to the computer running the task via Visual Basic 2010 (Microsoft Corporation, Redmond, Washington, USA). These experiments were video recorded from outside the booth by two GoPro Hero 8 Black cameras attached above the screen and on the side of the booth. Experiment 4 consisted of a visual fixation task using an eye-tracker (300 Hz; X300; Tobii AB, Stockholm, Sweden) connected to a laptop (Galleria GR2060RGF-T) with its software (Tobii Pro Lab v.1.181.37603), a method originally developed with humans, now widespread with other great ape species.^104^^,^^105^ Chimpanzees were 60-70 cm away from a screen (16:9; 1920 × 1080 px) placed behind the Plexiglas of the booth, with their head held straight using a grape juice-sucking system (described in Kano & Call^106^) involving a drip bag and tube inserted into a hole in the Plexiglas of the booth. The light of the room was turned off and the subject partially isolated from visual distractors by covering the side walls of the booth with black curtains (Figure S5).

#### Experiment 1: Disgust-related images

We tested the influence of disgust-related images (DIRTI) on six chimpanzees’ performance in the number ordering task. To do this, we selected free right DIRTI from Haberkamp et al.^60^ (https://zenodo.org/records/167037), and from free (Unplash) and paid (iStock, Adobe Stock) image platforms. We selected three conditions of disgust elicitors based on disgust ratings in humans^60^ and their relevance to chimpanzees: heterospecific animal carcasses (from other mammals, birds, amphibians, and marsupials, showing typical signs of decay such as tissue breakdown, presence of maggots, visible skeleton), disease-associated (e.g. mosquito, leech) or -mimicking (e.g. earthworm, slug) invertebrates, and rotten food (spoiled fruits and vegetables) (Figures S1–S3). Control image conditions were sleeping animals, invertebrates not associated with disease risk and edible food items, respectively. One session consisted of 59 trials of the number ordering task. It started with nine warm-up trials, followed by the full-screen presentation of a disgust-related or control image (1260 × 945 mm) for one second. This image was then followed by one test trial and four baseline trials, resulting in 10 image presentations per session. Each chimpanzee completed one randomized image session per condition per day. Each condition was repeated across three sessions per subject, and each session consisted of a different set of images (i.e. 3 sets of 10 different images per condition). In total, chimpanzees performed 108 sessions (3 sessions × 6 image conditions × 6 subjects).

#### Experiment 2: Pathogen-associated odors

Here, we tested whether pathogen-associated odors (PATOS) had a distracting effect on chimpanzee cognitive performance, using a paradigm similar to that used for DIRTI. Specifically, we used two volatile organic compounds previously employed in aversion research with humans and zebrafish^72^^,^^107^^,^^108^^,^^109^: cadaverine, associated with body decomposition; and butyric acid, associated with vomit and illness. Two control odors, limonene and water, were also included. Odors were delivered via a custom-built olfactory phenotyping device (“Smox”) placed beneath the touch screen and behind a thin grid. The device consisted of a 27 × 26 × 15 cm polycarbonate box containing a nebulizer (Omron NE-U100) with 6 mL of water or odor solution: 6 mL of water + 150 μL limonene (2.5% concentration) / 20 μL cadaverine (1,5-Diaminopentane) / butyric acid (both at 0.3% concentration) (all Tokyo Chemical Industry), and a small battery-powered fan on top (Figure 2B). Odor concentrations were chosen based on pilot tests with human experimenters to ensure they were detectable yet tolerable. Each session consisted of 50 trials of the number ordering task under continuous odor diffusion, starting upon the chimpanzee entering the booth; therefore, no warm-up trials were included. To maintain task engagement, we increased the reward’s nutritive value (i.e. piece of apple plus berries) and reduced the number of numerals shown (from 9 to 6). A maximum of two chimpanzees were tested per PATOS per day, with at least a four-hour interval (except for water) and the use of an air purifier (LEAF 320i) between sessions. Each chimpanzee completed three sessions per odor condition (72 sessions in total).

#### Experiment 3: Carcass versus snake images

We applied the same design as in experiment 1, using four image conditions: heterospecific animal carcasses (new set of images), snakes, and scrambled mosaics of both image types (Figure 1C). The scrambled mosaics were generated using WebMorph^110^ by dividing the original images into 12 × 9 squares. These mosaics preserve low-level visual features while disrupting the global structure of the image, a manipulation shown to significantly reduce the activation of pulvinar neurons in response to snake stimuli.^58^ The same six chimpanzees as in experiment 1 participated (Table S1), completing a total of 72 sessions (3 sessions × 4 image conditions × 6 subjects).

#### Experiment 4: Eye-tracking

We presented a total of 90 images to 8 chimpanzees ([10 images x 2 test categories x 3 sessions] + [10 images x 3 control categories x 1 session]). The test set included heterospecific animal carcasses and snakes (new sets of images), while the control sets featured live ungulates (deer), birds (pigeons) and tortoises, which are not known to be associated to specific threats to chimpanzees. Each image featured one single animal, full body from front, top or profile in a natural background environment. The images were positioned centrally within a grey horizontal slide, occupying 25% of the screen. Each session started with eye calibration, followed by the display of the first image (of one category) for five seconds. Following this, three fixation points, located in one corner of each slide (such as one cross, one number and one dot in the top right corner) would be presented one after another by switching via key press. Subsequently, the second image of the same category would be displayed for five seconds, followed by the relocation of the three fixation points to another corner of the slide, and this pattern would continue until the presentation of the tenth image, ending the session (Video S5). The same image order for each set was presented to the chimpanzees. For each picture slide, we defined three areas of interest (AOIs): 1) the animal, 2) the environment surrounding it, and 3) the grey area surrounding the picture frame (Figure S4). We considered three proxies of visual attention capture and retention, obtained via Tobii Pro Lab: 1) first fixation location, 2) proportion of fixations, and 3) attention score (i.e. the proportion of time looking at the animal versus elsewhere). We redid a session if it contained below 30% of fixations or if the subject left the experimental room, both happening in less than 10% of all sessions. A few exceptions were made: Pal’s fourth attempt at the “carcass” third session, which resulted in only 25% of fixations; Akira’s “snake” first and third sessions, where he left midway after several failed attempts; and Akira’s “deer” session, for which he never passed calibration. We therefore retained Pal’s final attempt at the “carcass” third session but excluded Akira’s “snake” first and third, and “deer” sessions. The low threshold (percentage of fixations) retained is voluntary and due to the prediction, associated with the task, that DIRTI elicit gaze avoidance.^14^ After data filtering based on these conditions, we obtained 47 recordings for the test sessions and 23 recordings for the control sessions.

Video S5. Chimpanzee Cleo performing the eye-tracking task under the tortoise condition (experiment 4) The session began with calibration using small moving icons paired with sounds, followed by a corner calibration. Each trial then consisted of a 5-s image presentation, after which corner calibration was repeated to decentralize gaze. This sequence was repeated until 10 images had been presented. Gaze plots illustrate fixations, with circle size reflecting fixation duration, and saccades (lines between circles).

### Quantification and statistical analysis

We analyzed how disgust- and fear-related sensory cues influenced chimpanzee cognitive processes using generalized linear mixed models (GLMMs), implemented via the glmmTMB package.^111^

In experiments 1-3, response variables were trial accuracy (binary; correct = 1, incorrect = 0), modeled with a binomial distribution; reaction time (continuous; latency to touch the first numeral); and latency to complete correct trials (continuous), both modeled with Gamma distributions. Only data from after the initial nine warm-up trials were included in analyses for experiments 1 and 3. We excluded trials with latencies below 300 ms (indicative of anticipatory responses or hands already on the screen) or above 2500 ms (suggesting distraction or disengagement). Control conditions in experiment 1 (images of animals sleeping, non-pathogenic invertebrates and edible food items) were tested for equivalence in success rates via a Chi-squared test before being grouped into a single control category (see results). Predictor variables included condition (i.e. the different images or odors), trial number (1-50; scaled and included in the accuracy models for experiments 1-3 and the reactivity and latency models for experiment 2), number of image exposure (1-10; scaled and included in the reactivity and latency models for experiments 1 and 3), and session number (1-3; included in the reactivity and latency models for experiment 2). Note that for the reactivity and latency models of experiments 1 and 3, only the trials immediately following image exposure were retained for data analysis. While the accuracy models account for potential longer-lasting effects of the images on cognition, the reactivity and latency models are designed to capture the immediate effect of image exposure. All models included a random intercept for subject identity to account for repeated measures on the same participants. In addition, models included a nested random effect of subjects within testing dates to account for potential variability across sessions; this nested effect was omitted from the accuracy model in experiment 1 because it complicated model fitting without improving fit.

In experiment 4, the response variables included the location of the first fixation (binary; animal vs. other); the proportion of fixations on the animal (continuous; bounded between 0 and 1) and the proportion of time spent looking at the animal (continuous; bounded between 0 and 1). Proportion data were modeled using beta distributions. There was one predictor variable: condition (image category) and three random effects: the slide number per session to account for variability in the image presented, subject identity and observation (row ID; for proportion models only).

Model comparisons were conducted using likelihood ratio tests (LRTs) from the lmtest package,^112^ comparing full models (including all predictors) against null models with only control variables. We also tested models with and without interactions of interest (e.g. condition and trial number). Model diagnostics were performed using the DHARMa package^113^ to check residual homogeneity, homoscedasticity, and zero-inflation; no violations of model assumptions were found, except for the Proportion of fixations model, which indicated a slight deviation from uniform residual distribution (Kolmogorov-Smirnov test: D = 0.055, *p* = 0.030). Despite this, the model provided the best fit among several alternatives (lower AIC, more stable estimates), and residual patterns did not suggest substantial model misspecification. We therefore retained this model for inference, while acknowledging minor limitations in model fit. For all retained models, we conducted pairwise post hoc comparisons among conditions using the emmeans package,^114^ applying Tukey correction (or Bonferroni correction for pre-selected contrasts in experiment 3). All analyses were conducted in R v.4.4.2.^115^
