# Supplementary material for: Dissecting disgust and fear in chimpanzees: From facilitation to disruption of cognitive processes
Source: iScience. 2026 Jul 29;29(8):116898. doi: 10.1016/j.isci.2026.116898 (PMC13451897; doi:10.1016/j.isci.2026.116898)
Supplement: Supplementary file 1 [file mmc1.pdf]

iScience, Volume *29*

Supplemental information

Dissecting disgust and fear in chimpanzees: From facilitation to disruption of cognitive processes

Cécile Sarabian, Andrew J.J. MacIntosh, André Gonçalves, Nobuyuki Kawai, and Ikuma Adachi

Table S1: **Participating subject details (related to STAR Methods).** Subjects were nine chimpanzees (*Pan troglodytes* spp.) from Kyoto University Primate Research Institute, involved in Experiments 1–4. Sex is given as m for males and f for females, and age is reported in years at the time of the study. Further details can be accessed on the Great Ape Information Network (GAIN) platform (https://shigen.nig.ac.jp/gain/) for which IDs and places of birth of the chimpanzees are provided.

Subject	Subspecies	Sex	Age	GAIN ID	Place of birth	Experiment
Ai	*P.t. verus*	F	44	0434	Wild-born	1–4
Akira	*P.t. verus*	M	44	0435	Wild-born	4
Ayumu	*P.t. verus*	M	20	0608	PRI	1–4
Chloe	*P.t. verus* x *P.t. troglodytes*	F	40	0441	Paris Zoo	1–4
Cleo	F2 hybrid x *P.t. verus*	F	20	0609	PRI	1–4
Gon	*P.t. verus*	M	54	0434	Wild-born	4
Pal	*P.t. verus*	F	20	0611	PRI	1–4
Pan	*P.t. verus*	F	36	0440	PRI	4
Pendesa	hybrid	F	43	0095	Japan Monkey Centre	1–3

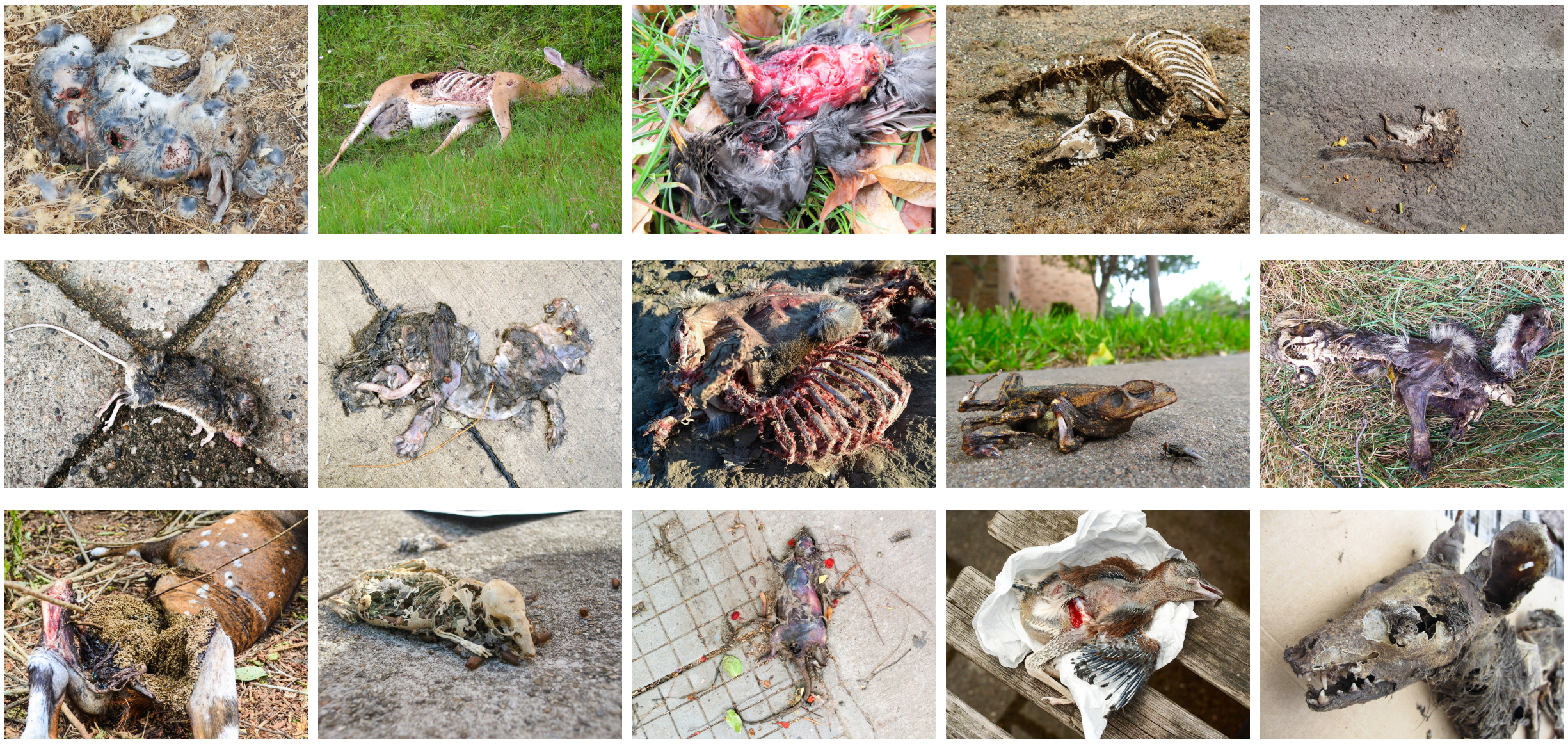

Fig. S1: **Animal carcass images used in Experiment 1 (related to STAR Methods).** Each row displays images from the same session. Those shown here are from the DIRTI database (https://zenodo.org/records/167037). Five additional images per session came from paid platforms and are not available for redistribution.

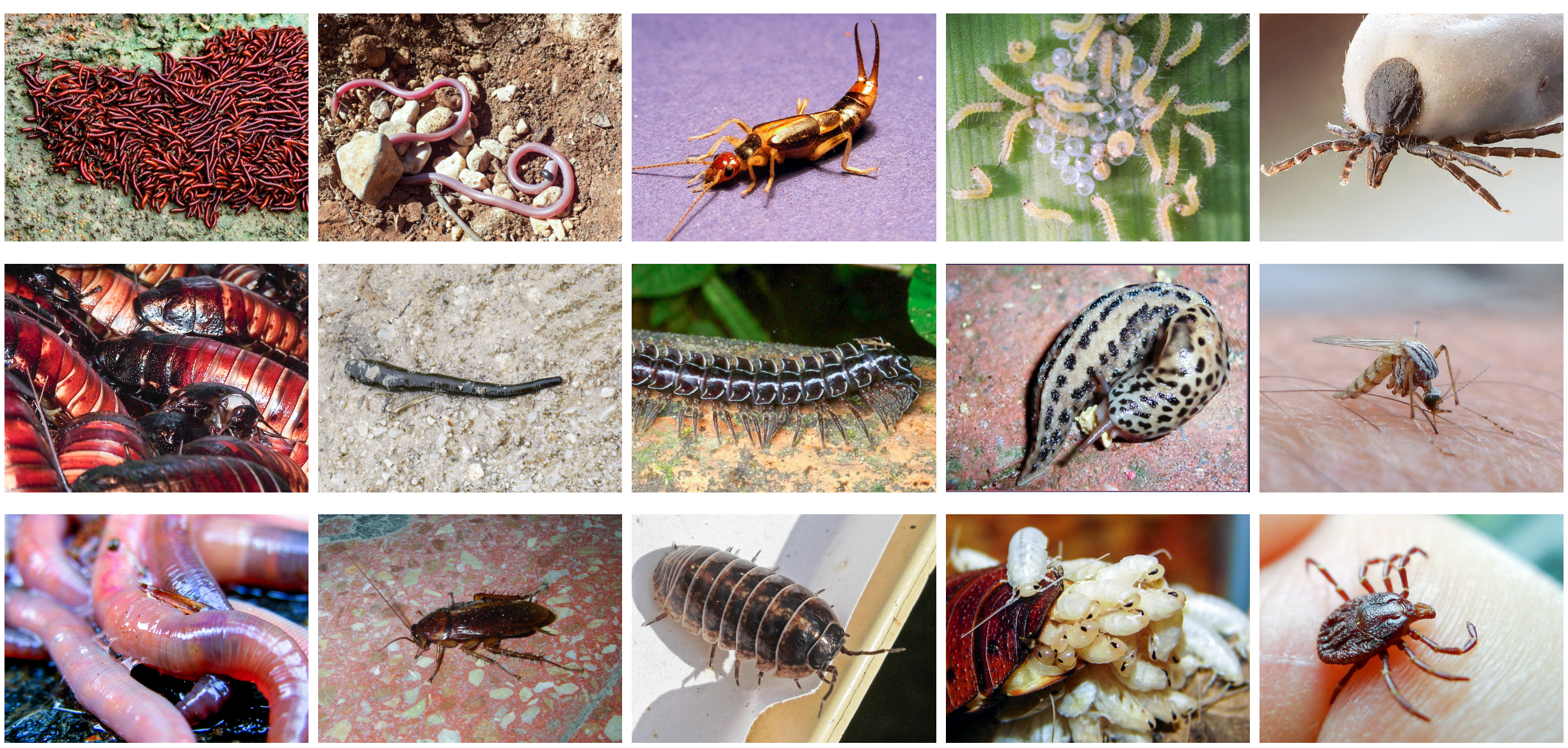

Fig. S2: **Disease-associated/pathogen-mimicking invertebrate images used in Experiment 1 (related to STAR Methods).** Each row displays images from the same session. Those shown here are from the DIRTI database (https://zenodo.org/records/167037). Five additional images per session came from paid platforms and are not available for redistribution.

Table S2: **Likelihood ratio tests comparing full models vs null and/or additive models for Experiments 1-4 (M1-4) for the different response variables (MX.1-3; related to STAR Methods).** Full models include predictors of interest or interactions between predictors. Null models contain only control predictors or the intercept. Additive models contain predictors of interest without interaction terms. Bold text denotes models that significantly outperformed their respective null or additive models. Significance levels: ***$p < 0.001$, **$p < 0.01$, *$p < 0.05$.

Statistical Model [X]	ΔLogLik	Δd.f.	X^2	p-value
**Experiment 1 (DIRTI)**				
**[M1.1] Accuracy**				
**Condition*Trial vs Trial**	**8.10**	**6**	**16.24**	**0.013***
Condition*Trial vs Condition + Trial	3.60	3	7.34	0.062
**Condition + Trial vs Trial**	**4.50**	**3**	**8.90**	**0.031***
[M1.2] Reactivity				
Condition*Exposure vs Exposure	5.10	6	10.27	0.114
**[M1.3] Latency**				
**Condition*Exposure vs Exposure**	**9.40**	**6**	**18.87**	**0.004****
**Condition*Exposure vs Condition + Exposure**	**7.00**	**3**	**13.97**	**0.003****
**Experiment 2 (PATOS)**				
[M2.1] Accuracy				
Odor*Trial*Session vs Trial + Session	6.10	13	12.25	0.507
[M2.2] Reactivity				
Odor*Trial*Session vs Trial + Session	9.00	13	17.12	0.194
**[M2.3] Latency**				
**Odor*Trial*Session vs Trial + Session**	**16.00**	**13**	**33.64**	**0.001****
**Odor*Trial*Session vs Odor + Trial + Session**	**15.00**	**10**	**31.37**	**< 0.001*****
**Experiment 3 (Carcass vs snake images)**				
[M3.1] Accuracy				
Condition*Image*Trial vs Trial	5.80	6	11.53	0.073
[M3.2] Reactivity				
Condition*Image*Exposure vs Exposure	4.10	6	8.21	0.223
**[M3.3] Latency**				
**Condition*Image*Exposure vs Exposure**	**7.10**	**6**	**14.27**	**0.027***
**Condition*Image*Exposure vs Cond + Image + Expo**	**6.90**	**4**	**13.77**	**0.008****
**Experiment 4 (Eye-tracking)**				
**[M4.1] First fixation location**				
**Condition vs intercept-only model**	**14.92**	**4**	**29.84**	**< 0.001*****
**[M4.2] Proportion of fixations**				
**Condition vs intercept-only model**	**32.68**	**4**	**65.37**	**< 0.001*****
**[M4.3] Proportion of time looking**				
**Condition vs intercept-only model**	**42.68**	**4**	**85.36**	**< 0.001*****

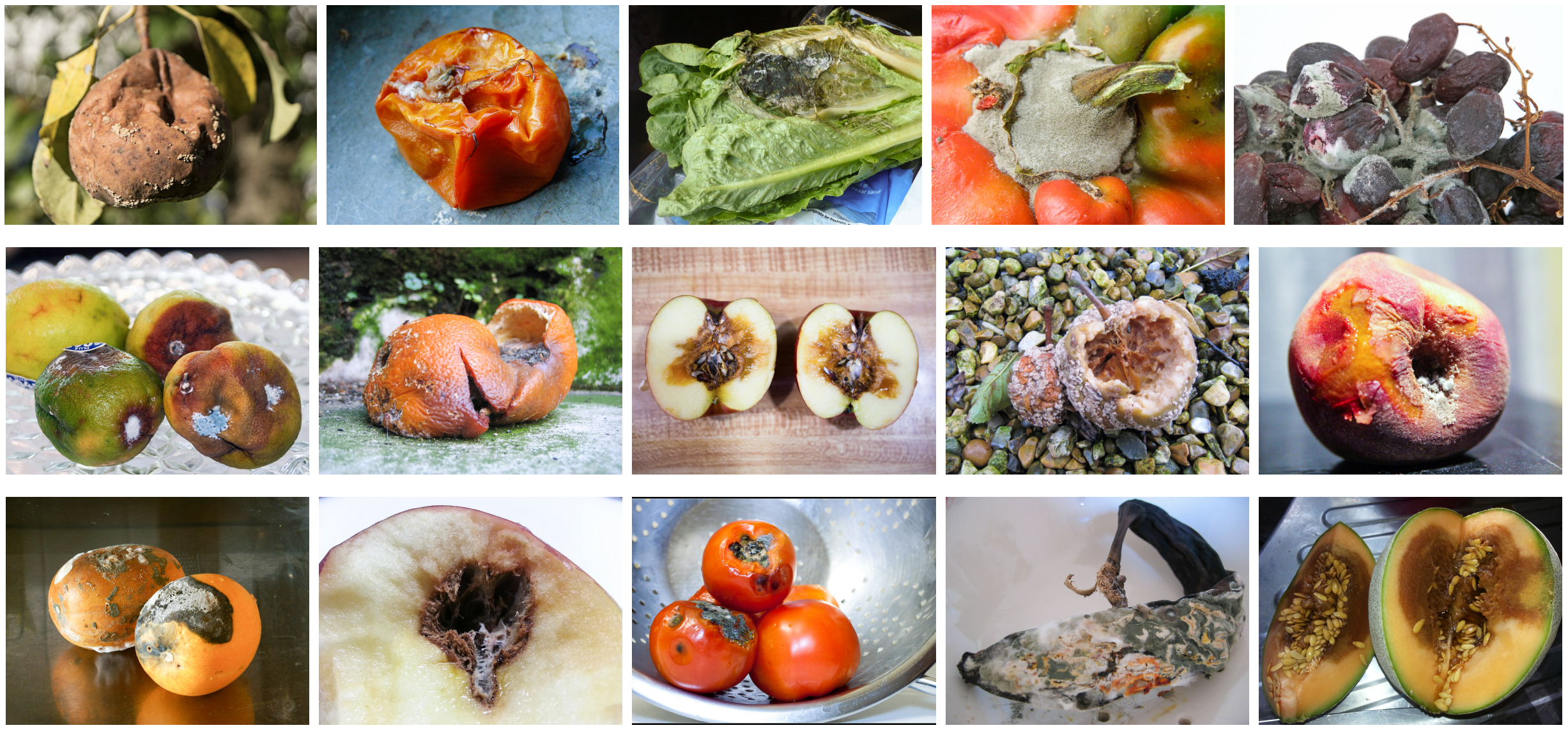

Fig. S3: **Rotten food images used in Experiment 1 (related to STAR Methods).** Each row displays images from the same session. Those shown here are from the DIRTI database (https://zenodo.org/records/167037). Five additional images per session came from paid platforms and are not available for redistribution.

Table S3: **Summary of GLMM results for predictors of interest in Experiment 1 (related to Figure 2).** The reference level is the pooled control image condition. Significant effects are highlighted in bold. Significance levels: $***p < 0.001$, $**p < 0.01$, $*p < 0.05$.

Model	Predictor	Estimate (β)	SE	z	p-value
[M1.1] Accuracy	(Intercept)	1.691	0.166	10.17	$< 2e - 16 * **$
	**Carcass**	**-0.244**	**0.100**	**-2.44**	**0.015***
	Invertebrate	0.105	0.109	0.96	0.335
	Rotten food	-0.096	0.103	-0.93	0.354
	Trial number	0.037	0.037	0.99	0.323
[M1.3] Latency	(Intercept)	8.312	0.051	164.43	$< 2e - 16 * **$
	Carcass	-0.008	0.019	-0.44	0.657
	Invertebrate	-0.021	0.018	-1.17	0.241
	Rotten food	0.030	0.018	1.64	0.102
	Exposure	-0.002	0.006	-0.29	0.770
	Carcass*Exposure	0.015	0.013	1.16	0.247
	Invertebrate*Exposure	0.003	0.013	0.23	0.816
	**Rotten food*Exposure**	**-0.041**	**0.013**	**-3.13**	**0.002****

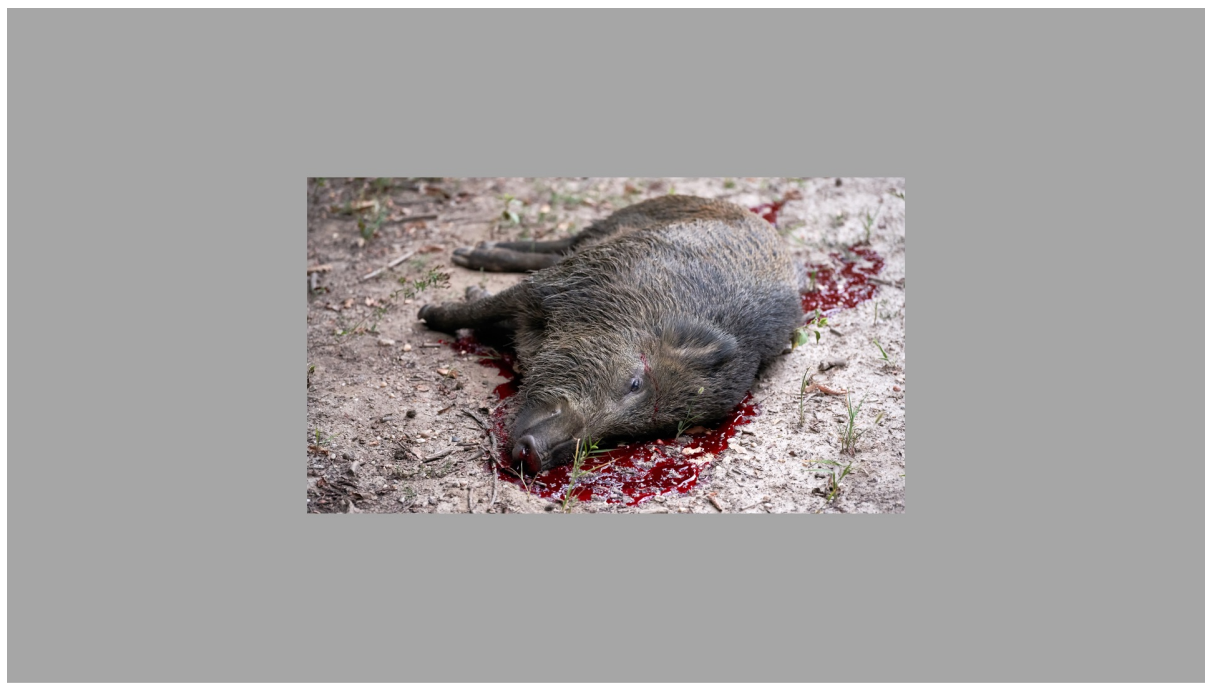

(a) First carcass image of session 1

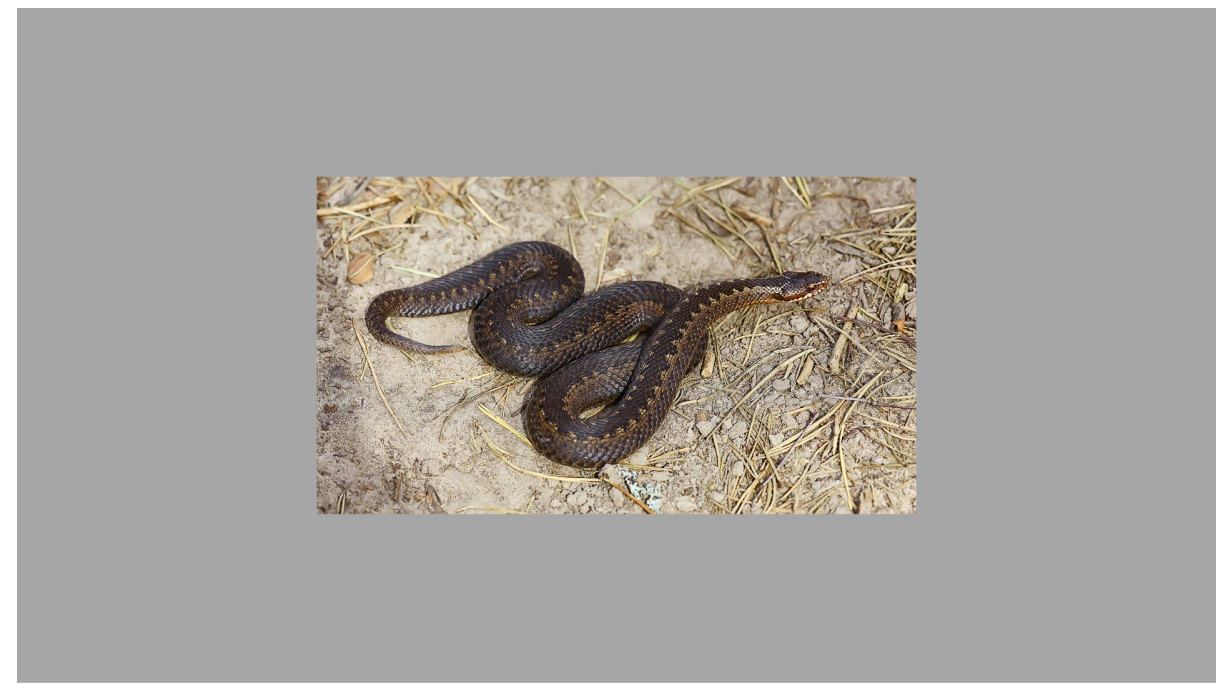

(b) First snake image of session 1

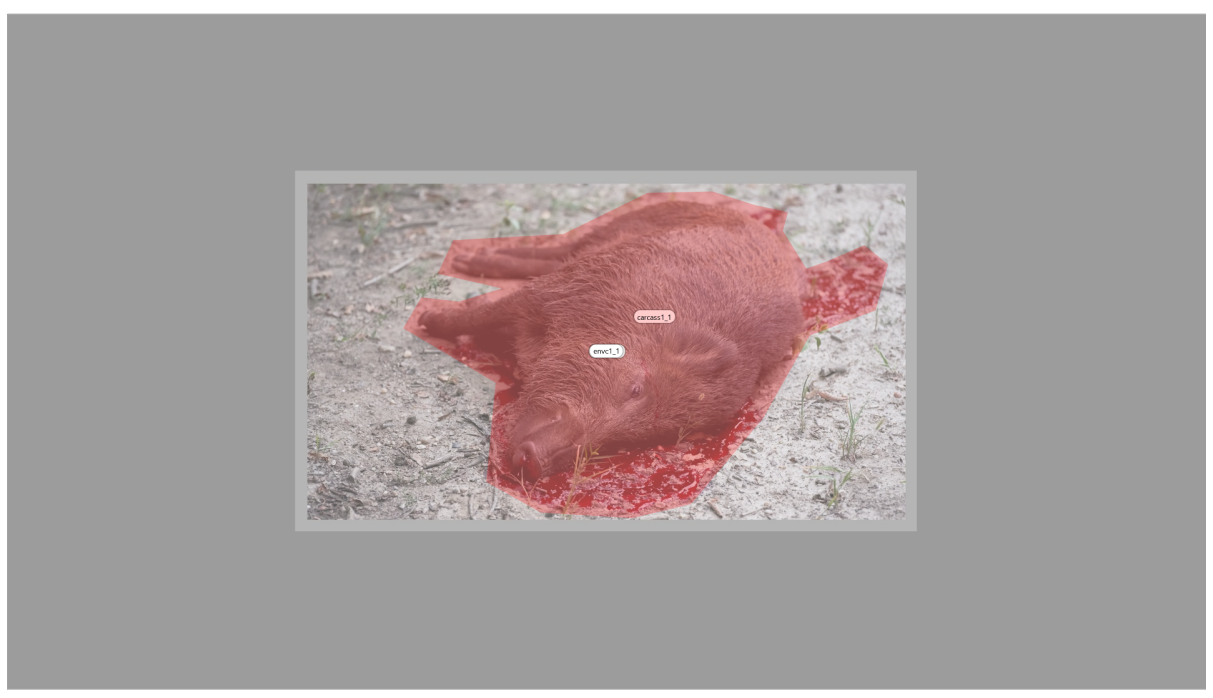

(c) Corresponding areas of interest

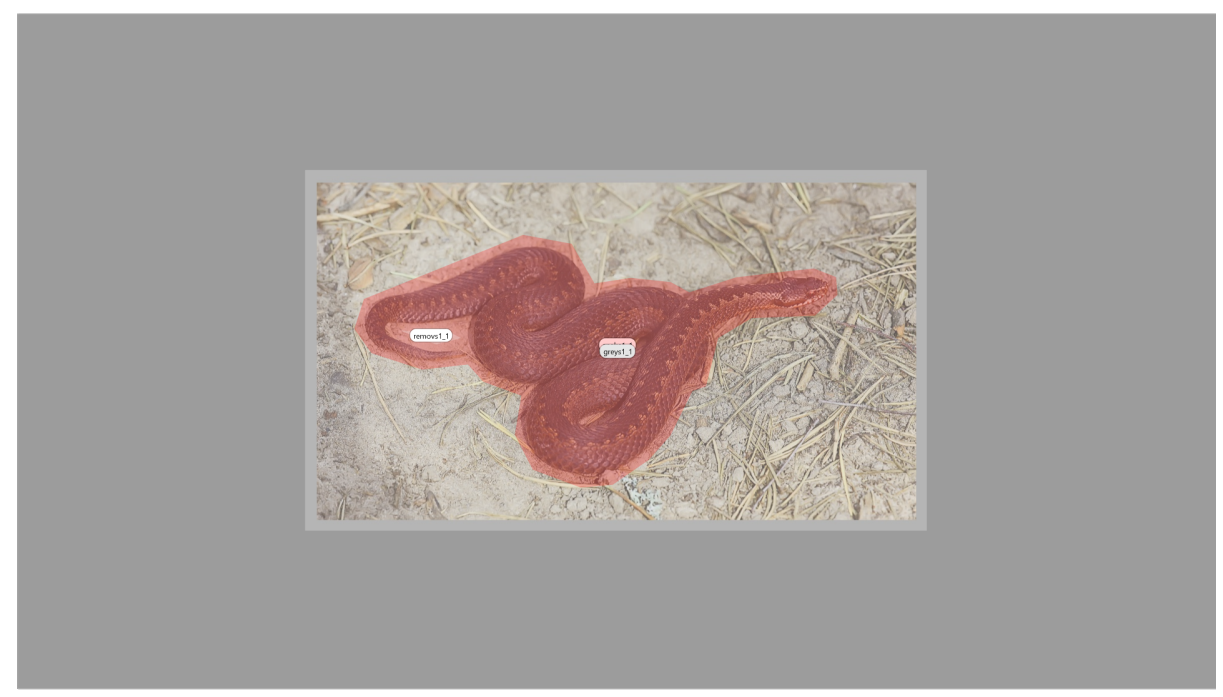

(d) Corresponding areas of interest

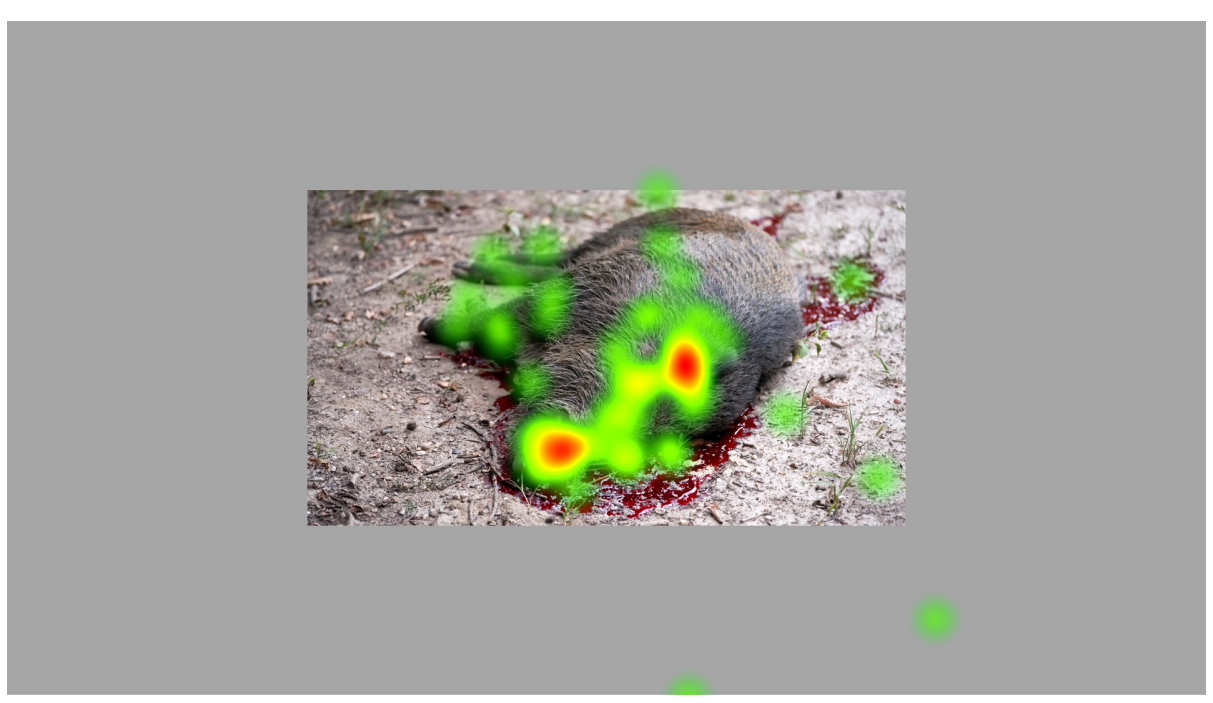

(e) Corresponding heat map

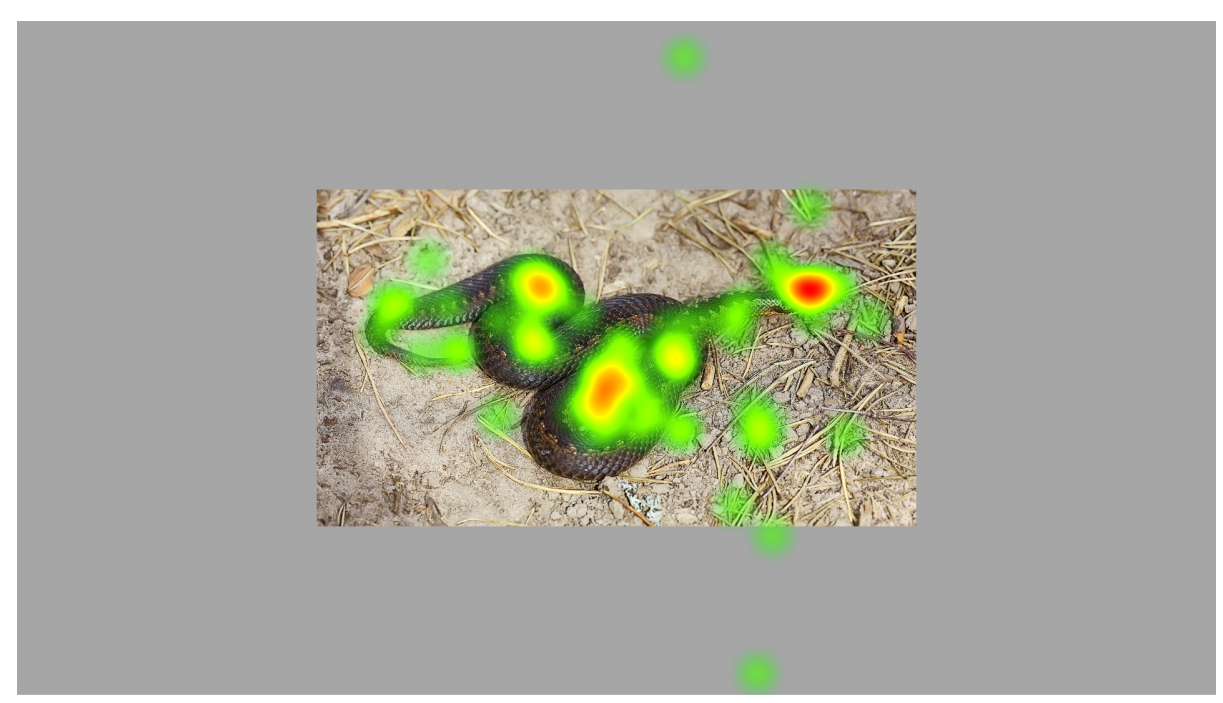

(f) Corresponding heat map

Fig. S4: **Areas of interest considered in Experiment 4 (related to STAR Methods).** These included the animal, its environment and the grey area surrounding the picture frame, as illustrated in (c) and (d) for images (a) and (b), respectively. The heat maps (e) and (f) represent the corresponding fixation counts for all chimpanzees; the “hotter” areas are where participants fixated more often.

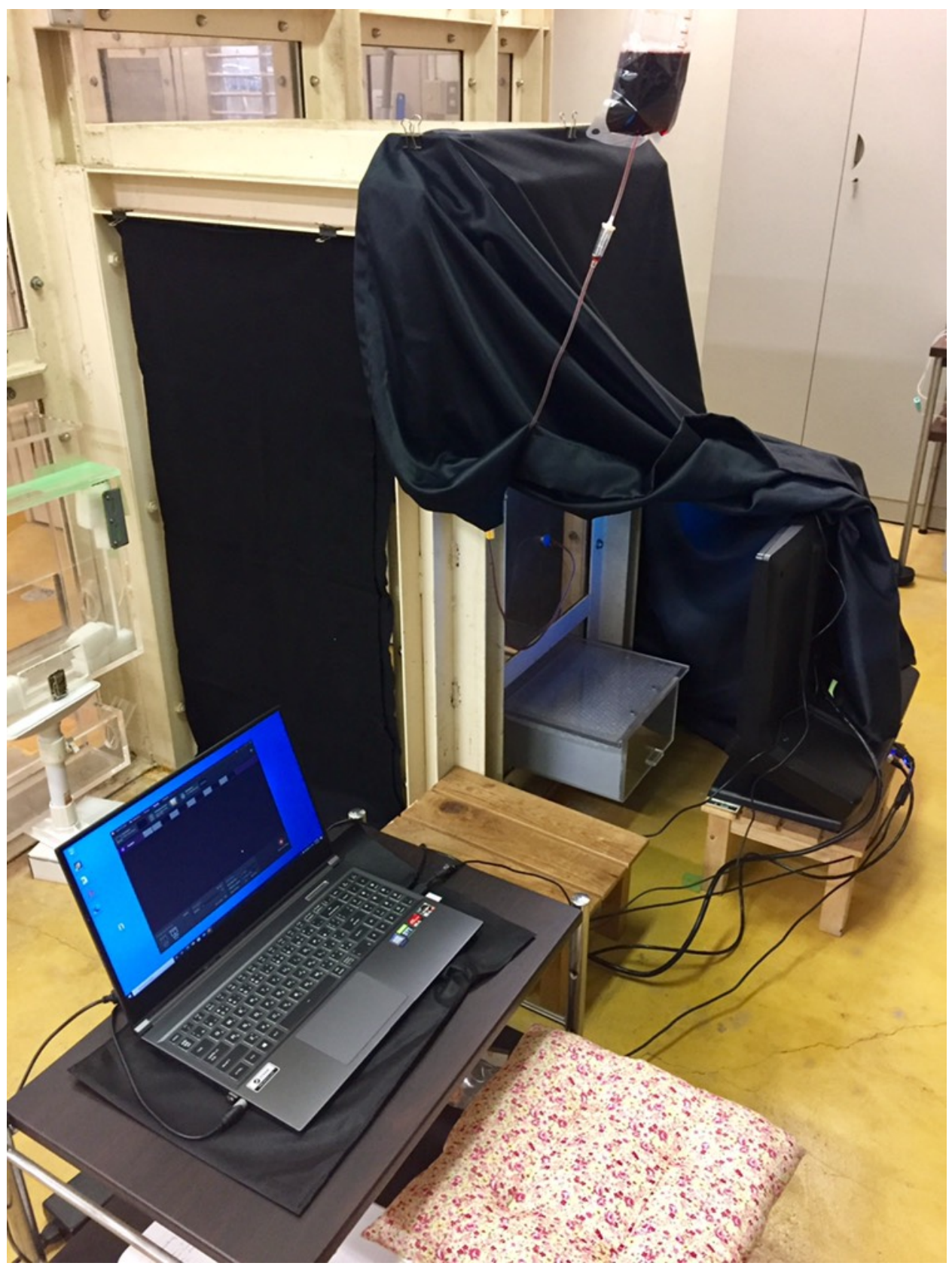

Fig. S5: **Experimental setup for Experiment 4 (related to STAR Methods).** Chimpanzees were positioned 60–70 cm from the monitor screen with their head stabilized using a grape juice–sucking system (drip bag and tube inserted through the Plexiglas panel). The testing room was darkened, and side walls of the booth were covered with black curtains to reduce visual distractions.

Table S4: **Estimated marginal means and pairwise comparisons of accuracy and latency across image conditions in Experiment 1 (related to Figure 2).** For latency, analyses were performed at min, mid, and high image exposure. Image codes: Ctrl = Control; C = Carcass; I = Invertebrate; RF = Rotten food. Pairwise comparisons are Tukey-adjusted. Results for accuracy are shown on the logit scale, and those for latency on the log scale. Significant effects are highlighted in bold. Significance levels: ***p < 0.001, **p < 0.01, *p < 0.05.

Model	Expo	Cond	EMM (95% CI)	Contrast	β	SE	z	p-value
[M1.1] Accuracy		Ctrl	1.69 [1.37, 2.02]	-	-	-	-	-
		C	1.45 [1.10, 1.80]	Ctrl - C	0.244	0.100	2.44	0.070
		I	1.80 [1.44, 2.16]	Ctrl - I	-0.105	0.109	-0.96	0.770
		RF	1.60 [1.24, 1.95]	Ctrl - RF	0.096	0.103	0.93	0.791
				**C - I**	**-0.349**	**0.127**	**-2.74**	**0.031***
				C - RF	-0.149	0.123	-1.21	0.622
				I - RF	0.200	0.130	1.54	0.412
[M1.3] Latency	Min	Ctrl	8.31 [8.21, 8.42]	-	-	-	-	-
		C	8.28 [8.17, 8.39]	Ctrl - C	0.033	0.028	1.18	0.641
		I	8.29 [8.18, 8.40]	Ctrl - I	0.026	0.027	0.96	0.773
		RF	8.41 [8.30, 8.52]	**Ctrl - RF**	**-0.096**	**0.029**	**-3.34**	**0.005****
				C - I	-0.006	0.034	-0.19	0.998
				**C - RF**	**-0.128**	**0.035**	**-3.69**	**0.001****
				**I - RF**	**-0.122**	**0.035**	**-3.53**	**0.002****
	Mid	Ctrl	8.31 [8.21, 8.41]	-	-	-	-	-
		C	8.30 [8.20, 8.41]	Ctrl - C	0.008	0.019	0.44	0.971
		I	8.29 [8.19, 8.39]	Ctrl - I	0.021	0.018	1.17	0.644
		RF	8.34 [8.24, 8.44]	Ctrl - RF	-0.030	0.019	-1.64	0.359
				C - I	0.013	0.023	0.58	0.939
				C - RF	-0.039	0.023	-1.69	0.331
				I - RF	-0.052	0.022	-2.30	0.098
	High	Ctrl	8.31 [8.21, 8.41]	-	-	-	-	-
		C	8.32 [8.22, 8.43]	Ctrl - C	-0.016	0.028	-0.55	0.947
		I	8.29 [8.18, 8.40]	Ctrl - I	0.017	0.027	0.61	0.929
		RF	8.27 [8.17, 8.38]	Ctrl - RF	0.034	0.027	1.27	0.584
				C - I	0.032	0.034	0.94	0.783
				C - RF	0.050	0.034	1.46	0.460
				I - RF	0.017	0.033	0.53	0.953

Table S5: **Summary of GLMM results for predictors of interest in Experiment 2 (related to Figure 2).** The reference odor is water. Significant effects are highlighted in bold. Significance levels: ***p < 0.001, **p < 0.01, *p < 0.05.

Model	Predictor	Estimate (β)	SE	z	p-value
[M2.3] Latency	(Intercept)	7.946	0.047	168.10	$< 2e-16 ***$
	Butyric acid	-0.095	0.054	-1.77	0.077
	Cadaverine	-0.054	0.051	-1.04	0.297
	Limonene	-0.036	0.051	-0.70	0.487
	**Trial**	**0.073**	**0.022**	**3.29**	**< 0.001*** **
	Session	0.031	0.017	1.85	0.064
	**Butyric acid*Trial**	**-0.126**	**0.033**	**-3.78**	**< 0.001*** **
	**Cadaverine*Trial**	**-0.119**	**0.033**	**-3.65**	**< 0.001*** **
	Limonene*Trial	-0.060	0.032	-1.89	0.059
	**Butyric acid*Session**	**0.068**	**0.025**	**2.75**	**0.006** **
	Cadaverine*Session	0.030	0.024	1.25	0.211
	Limonene*Session	0.027	0.024	1.11	0.266
	**Trial*Session**	**-0.041**	**0.010**	**-3.94**	**< 0.001*** **
	**Butyric acid*Trial*Session**	**0.048**	**0.015**	**3.12**	**< 0.001*** **
	**Cadaverine*Trial*Session**	**0.056**	**0.015**	**3.60**	**< 0.001*** **
	Limonene*Trial*Session	0.021	0.015	1.40	0.160

Table S6: **Estimated marginal means and pairwise comparisons from the GLMM testing the effects of odor, trial number, and session on latency in Experiment 2 (related to Figure 2).** Analyses were performed at early, mid, and high trial numbers. Odor codes: W = Water; BA = Butyric acid; C = Cadaverine; L = Limonene. Pairwise comparisons are Tukey-adjusted. Significant effects are highlighted in bold. Significance levels: ***$p < 0.001$, **$p < 0.01$, *$p < 0.05$.

Session – Trial	Condition	EMM (95% CI)	Contrast	β	SE	z	p-value
1 – Early	W	7.93 [7.84, 8.02]	-	-	-	-	-
	BA	8.01 [7.92, 8.11]	W - BA	-0.083	0.051	-1.63	0.364
	C	8.02 [7.92, 8.11]	W - C	-0.085	0.049	-1.72	0.315
	L	7.95 [7.86, 8.04]	W - L	-0.020	0.048	-0.41	0.977
			BA - C	-0.002	0.052	-0.04	1.000
			BA - L	0.063	0.051	1.25	0.598
			C - L	0.065	0.049	1.33	0.547
1 – Mid	W	7.98 [7.91, 8.06]	-	-	-	-	-
	BA	7.96 [7.88, 8.04]	W - BA	0.029	0.035	0.82	0.845
	C	7.96 [7.89, 8.04]	W - C	0.020	0.033	0.62	0.926
	L	7.97 [7.90, 8.05]	W - L	0.014	0.033	0.44	0.972
			BA - C	-0.008	0.035	-0.23	0.996
			BA - L	-0.014	0.035	-0.41	0.977
			C - L	-0.006	0.033	-0.18	0.998
1 – High	W	8.04 [7.95, 8.13]	-	-	-	-	-
	BA	7.90 [7.81, 8.00]	**W - BA**	**0.138**	**0.050**	**2.75**	**0.031***
	C	7.91 [7.82, 8.01]	**W - C**	**0.124**	**0.048**	**2.57**	**< 0.05***
	L	7.99 [7.90, 8.08]	W - L	0.048	0.047	1.02	0.740
			BA - C	-0.014	0.051	-0.28	0.993
			BA - L	-0.090	0.050	-1.80	0.273
			C - L	-0.076	0.048	-1.58	0.388
2 – Early	W	8.00 [7.91, 8.09]	-	-	-	-	-
	BA	8.14 [8.04, 8.24]	W - BA	-0.140	0.055	-2.56	0.051
	C	8.03 [7.93, 8.12]	W - C	-0.024	0.051	-0.48	0.964
	L	8.14 [8.05, 8.23]	**W - L**	**-0.139**	**0.051**	**-2.70**	**0.035***
			BA - C	0.116	0.055	2.10	0.153
			BA - L	0.001	0.056	0.03	1.000
			C - L	-0.115	0.052	-2.21	0.122
2 – Mid	W	7.99 [7.91, 8.06]	-	-	-	-	-
	BA	8.04 [7.95, 8.12]	W - BA	-0.047	0.038	-1.25	0.596
	C	8.00 [7.92, 8.07]	W - C	-0.006	0.035	-0.18	0.998
	L	8.01 [7.94, 8.09]	W - L	-0.025	0.035	-0.70	0.898
			BA - C	0.041	0.039	1.06	0.716
			BA - L	0.023	0.039	0.58	0.938
			C - L	-0.018	0.036	-0.51	0.957

Session – Trial	Condition	EMM (95% CI)	Contrast	β	SE	z	p-value
2 – High	W	7.98 [7.89, 8.07]	-	-	-	-	-
	BA	7.93 [7.83, 8.04]	W - BA	0.045	0.056	0.80	0.854
	C	7.97 [7.87, 8.06]	W - C	0.012	0.050	0.23	0.996
	L	7.89 [7.79, 7.99]	W - L	0.088	0.051	1.71	0.318
			BA - C	-0.033	0.057	-0.58	0.939
			BA - L	0.043	0.058	0.75	0.876
			C - L	0.076	0.053	1.45	0.471
3 – Early	W	8.13 [8.04, 8.22]	-	-	-	-	-
	BA	8.18 [8.09, 8.28]	W - BA	-0.053	0.051	-1.03	0.733
	C	8.09 [7.99, 8.18]	W - C	0.045	0.052	0.87	0.823
	L	8.11 [8.01, 8.21]	W - L	0.018	0.054	0.33	0.987
			BA - C	0.097	0.054	1.80	0.274
			BA - L	0.071	0.056	1.26	0.589
			C - L	-0.027	0.057	-0.47	0.965
3 – Mid	W	8.05 [7.97, 8.12]	-	-	-	-	-
	BA	8.15 [8.07, 8.23]	**W - BA**	**-0.107**	**0.035**	**-3.04**	**0.013***
	C	8.08 [8.01, 8.16]	W - C	-0.038	0.035	-1.07	0.708
	L	8.08 [8.00, 8.16]	W - L	-0.037	0.035	-1.03	0.731
			BA - C	0.069	0.037	1.88	0.238
			BA - L	0.070	0.037	1.91	0.224
			C - L	0.001	0.037	0.04	1.000
3 – High	W	7.96 [7.87, 8.05]	-	-	-	-	-
	BA	8.12 [8.03, 8.22]	**W - BA**	**-0.160**	**0.051**	**-3.12**	**< 0.01****
	C	8.08 [7.98, 8.18]	W - C	-0.119	0.052	-2.29	0.102
	L	8.05 [7.96, 8.15]	W - L	-0.090	0.051	-1.76	0.294
			BA - C	0.041	0.054	0.76	0.875
			BA - L	0.070	0.054	1.31	0.558
			C - L	0.029	0.054	0.53	0.951

Table S7: **Summary of GLMM results for predictors of interest in Experiment 3 (related to Figure 2).** The reference condition is control and the reference image carcass. Significant effects are highlighted in bold. Significance levels: ***$p < 0.001$, **$p < 0.01$, *$p < 0.05$.

Model	Predictor	Estimate (β)	SE	z	p-value
[M3.3] Latency	(Intercept)	8.251	0.064	129.24	$< 2e-16***$
	Condition:Test	0.014	0.022	0.66	0.506
	Image:Snake	0.003	0.022	0.12	0.903
	Exposure	-0.009	0.011	-0.75	0.452
	Test*Snake	-0.025	0.030	-0.81	0.417
	Test*Exposure	0.014	0.016	0.91	0.362
	Snake*Exposure	0.005	0.016	0.33	0.744
	**Test*Snake*Exposure**	**-0.057**	**0.022**	**-2.59**	**< 0.01****

Table S8: **Estimated marginal means and pairwise comparisons of latency across image types (carcass vs. snake) and conditions (test vs. control) in Experiment 3 (related to Figure 2).** Codes: C = Carcass; Ctrl = Control; S = Snake; T = Test. Comparisons of interest were selected and adjusted using the Bonferroni method. Significant effects are highlighted in bold. Significance levels: ***p < 0.001, **p < 0.01, *p < 0.05.

Exposure	Condition	EMM (95%)	Contrast	β	SE	z	p-value
Min	C*Ctrl	8.26 [8.13, 8.39]	-	-	-	-	-
	C*T	8.26 [8.13, 8.39]	C*Ctrl - S*Ctrl	0.005	0.033	0.16	1.000
	S*Ctrl	8.26 [8.13, 8.39]	C*T - S*T	-0.059	0.033	-1.81	0.279
	S*T	8.32 [8.19, 8.45]	C*Ctrl - C*T	0.008	0.033	0.24	1.000
			S*Ctrl - S*T	-0.057	0.032	-1.76	0.316
Mid	C*Ctrl	8.25 [8.13, 8.38]	-	-	-	-	-
	C*T	8.27 [8.14, 8.39]	C*Ctrl - S*Ctrl	-0.003	0.022	-0.12	1.000
	S*Ctrl	8.25 [8.13, 8.38]	C*T - S*T	0.022	0.022	0.99	1.000
	S*T	8.24 [8.12, 8.37]	C*Ctrl - C*T	-0.014	0.022	-0.67	1.000
			S*Ctrl - S*T	0.010	0.022	0.46	1.000
High	C*Ctrl	8.24 [8.11, 8.37]	-	-	-	-	-
	C*T	8.27 [8.15, 8.40]	C*Ctrl - S*Ctrl	-0.011	0.033	-0.32	1.000
	S*Ctrl	8.25 [8.12, 8.38]	**C*T - S*T**	**0.102**	**0.032**	**3.17**	**0.006****
	S*T	8.17 [8.04, 8.30]	C*Ctrl - C*T	-0.037	0.032	-1.14	1.000
			S*Ctrl - S*T	0.076	0.033	2.28	0.090

Table S9: **Summary of GLMM results for predictors of interest in Experiment 4 (related to Figure 3).** The reference condition is carcass. Significant effects are highlighted in bold. Significance levels: ***p < 0.001, **p < 0.01, *p < 0.05.

Model	Predictor	Estimate (β)	SE	z	p-value
[M4.1] First fixation location	(Intercept)	1.411	0.244	5.78	7.6e-9***
	Snake	0.001	0.237	0.01	0.995
	**Tortoise**	**-0.808**	**0.299**	**-2.70**	**0.007****
	**Pigeon**	**-1.387**	**0.298**	**-4.66**	**< 0.001*****
	**Deer**	**-0.854**	**0.330**	**-2.59**	**< 0.01****
[M4.2] Proportion of fixations	(Intercept)	0.522	0.106	4.94	7.9e-7***
	Snake	0.032	0.061	0.526	0.599
	**Tortoise**	**-0.193**	**0.096**	**-2.01**	**0.044***
	**Pigeon**	**-0.453**	**0.090**	**-5.05**	**< 0.001*****
	**Deer**	**-0.658**	**0.095**	**-6.90**	**< 0.001*****
[M4.3] Proportion of time looking	(Intercept)	0.723	0.131	5.54	3.1e-8***
	**Snake**	**0.157**	**0.074**	**2.12**	**0.034***
	**Tortoise**	**-0.247**	**0.115**	**-2.15**	**0.032***
	**Pigeon**	**-0.565**	**0.108**	**-5.25**	**< 0.001*****
	**Deer**	**-0.787**	**0.115**	**-6.83**	**< 0.001*****

Table S10: **Estimated marginal means and pairwise comparisons of visual attention across image conditions in Experiment 4 (related to Figure 3).** Visual attention is represented by the first fixation location, proportion of fixations, and proportion of time spent looking. Image codes: C = Carcass; D = Deer; P = Pigeon; S = Snake; T = Tortoise. Pairwise comparisons are Tukey-adjusted. Significance levels: ***p < 0.001, **p < 0.01, *p < 0.05.

Model	Cond	EMM (95% CI)	Contrast	β	SE	z	p-value
[M4.1] First fixation	C	1.41 [0.93, 1.89]	-	-	-	-	-
	D	0.56 [-0.10, 1.21]	C - D	0.854	0.330	2.59	0.097
	P	0.02 [-0.57, 0.62]	**C - P**	**1.387**	**0.298**	**4.66**	**< 0.001*** **
	S	1.41 [0.92, 1.90]	C - S	-0.001	0.237	-0.01	1.000
	T	0.60 [0.00, 1.20]	C - T	0.808	0.299	2.70	0.069
			S - D	0.856	0.333	2.57	0.101
			**S - P**	**1.388**	**0.303**	**4.58**	**< 0.001*** **
			S - T	0.809	0.305	2.66	0.079
			D - P	0.533	0.363	1.47	1.000
			D - T	-0.046	0.366	-0.13	1.000
			P - T	-0.579	0.336	-1.72	0.853
[M4.2] Prop fixations	C	0.52 [0.31, 0.73]	-	-	-	-	-
	D	-0.14 [-0.39, 0.12]	**C - D**	**0.658**	**0.095**	**6.90**	**< 0.001*** **
	P	0.07 [-0.18, 0.31]	**C - P**	**0.453**	**0.090**	**5.05**	**< 0.001*** **
	S	0.55 [0.34, 0.76]	C - S	-0.032	0.061	-0.53	1.000
	T	0.33 [0.08, 0.58]	C - T	0.193	0.096	2.01	0.260
			**S - D**	**0.690**	**0.096**	**7.18**	**< 0.001*** **
			**S - P**	**0.485**	**0.091**	**5.31**	**< 0.001*** **
			S - T	0.225	0.097	2.31	0.142
			D - P	-0.205	0.103	-1.99	0.272
			**D - T**	**-0.466**	**0.109**	**-4.28**	**< 0.001*** **
			P - T	-0.261	0.105	-2.49	0.092
[M4.3] Prop time	C	0.72 [0.47, 0.98]	-	-	-	-	-
	D	-0.06 [-0.37, 0.25]	**C - D**	**0.787**	**0.115**	**6.83**	**< 0.001*** **
	P	0.16 [-0.14, 0.46]	**C - P**	**0.565**	**0.108**	**5.25**	**< 0.001*** **
	S	0.88 [0.62, 1.14]	C - S	-0.157	0.074	-2.12	0.210
	T	0.48 [0.17, 0.79]	C - T	0.247	0.115	2.15	0.319
			**S - D**	**0.944**	**0.117**	**8.08**	**< 0.001*** **
			**S - P**	**0.722**	**0.110**	**6.55**	**< 0.001*** **
			**S - T**	**0.404**	**0.118**	**3.44**	**0.005** **
			D - P	-0.222	0.125	-1.78	0.386
			**D - T**	**-0.539**	**0.132**	**-4.10**	**< 0.001*** **
			P - T	-0.318	0.126	-2.53	0.085
